# Discrimination of Surface Topographies Created by Two-Stage Process by Means of Multiscale Analysis

**DOI:** 10.3390/ma14227044

**Published:** 2021-11-20

**Authors:** Tomasz Bartkowiak, Karol Grochalski, Bartosz Gapiński, Michał Wieczorowski

**Affiliations:** 1 Intelligent Machines Laboratory, Institute of Mechanical Technology, Poznan University of Technology, Piotrowo 3, PL-60965 Poznan, Poland; 2 Division of Metrology and Measurement Systems, Institute of Mechanical Technology, Poznan University of Technology, Piotrowo 3, PL-60965 Poznan, Poland; karol.grochalski@put.poznan.pl (K.G.); bartosz.gapinski@put.poznan.pl (B.G.); michal.wieczorowski@put.poznan.pl (M.W.)

**Keywords:** discrimination, surface texture, mass finishing, multiscale, hot rolling, roughness

## Abstract

The fundamental issue in surface metrology is to provide methods that can allow the establishment of correlations between measured topographies and performance or processes, or that can discriminate confidently topographies that are processed or performed differently. This article presents a set of topographies from two-staged processed steel rings, measured with a 3D contact profilometer. Data were captured individually from four different regions, namely the top, bottom, inner, and outer surfaces. The rings were manufactured by drop forging and hot rolling. Final surface texture was achieved by mass finishing with spherical ceramic media or cut wire. In this study, we compared four different multiscale methods: sliding bandpass filtering, three geometric length- and area-scale analyses, and the multiscale curvature tensor approach. In the first method, ISO standard parameters were evaluated as a function of the central wavelength and bandwidth for measured textures. In the second and third method, complexity and relative length and area were utilized. In the last, multiscale curvature tensor statistics were calculated for a range of scales from the original sampling interval to its forty-five times multiplication. These characterization parameters were then utilized to determine how confident we can discriminate (through F-test) topographies between regions of the same specimen and between topographies resulting from processing with various technological parameters. Characterization methods that focus on the geometrical properties of topographic features allowed for discrimination at the finest scales only. Bandpass filtration and basic height parameters Sa and Sq proved to confidently discriminate against all factors at all three considered bandwidths.

## 1. Introduction

The objective of this paper is to demonstrate the use of three different multiscale methods to discriminate between topographies that were created by two stage formation: hot rolling and mass finishing. The term “multiscale” analysis is used in this study to emphasize that the surface is studied at multiple scales of observation or calculation [1]. This type of analysis adds extra value and reduces costs in design of products and processes by providing better understanding of the relations with processing or with performance and topographies.

From the quality control perspective, it is essential to be able to differentiate or distinguish between surfaces that perform and were fabricated, modified, or treated differently. This ability is called discrimination, and thanks to multiscale analysis, it becomes possible to identify what surface characterization techniques, relating parameters, and scales are the most convenient at discerning topographies. The idea comes from the fact that topographic features of certain sizes and shapes that are signature of particular manufacturing process can be best discernible at a certain scale or scales of observation [2,3,4]. The hereby presented literature review focuses on the application of different multiscale analyses into the discrimination problems for surfaces.

Bigerelle et al. [5] studied the ability to discriminate the surface roughness of plastic parts created by injection molding, concentrating on four processing parameters. In that research, they focused on standard height parameters calculated as a function of evaluation length and the degree of polynomial fitted for roughness data. They found the most convenient combination of the roughness characterization and data processing parameter, as well as the scale for discrimination of the technological parameters and determined the scale at which each process leaves its characteristic surface signature.

Geometric multiscale method can be successfully applied to discern manufacturing conditions. The area-scale method has proven to be successful in discriminating grinding parameters and scales where they can be distinguished, and using length-scale for showing the grinding direction, on polyethylene ski bases, textured for achieving the best performance [6]. Multiscale characterization based on the area-scale method was employed to discriminate surface topographies, of pharmaceutical excipient compacts with different compositions and particle sizes. Relative area and fractal complexity helped to distinguish for scales of 1000 µm^2^, when the F-test was applied. This could not be achieved using conventional parameters [7,8].

Brown et al. studied FDM samples treated with acetone vapor [9,10]. Samples were discriminated using the area-scale method and conventional analysis. It was shown that at the scale finer than or approximately equal to the deposition layer thickness, multiscale outperformed the standard analysis by more than two times. In experimental work on fracture mechanics, confident distinguishing was achieved for yttria-stabilized tetragonal zirconia [11] and graphite electrodes [8] using area-scale analysis.

Another method utilized the wavelets theorem [12]. In that study, a multiscale decomposition involved using a continuous wavelet transform (CWT), which provided the multiscale transfer function of the surface topography, as measured by a white light interferometer, by the last stage manufacturing process. The ranking of the transfer function with respect to grit size varied with scale. The topographies created with various processing parameters were confidently distinguished. This facilitated the understanding of the tribological mechanisms that governed the process.

A large area of application of discrimination techniques is in archaeological science. Stemp created plots of the logarithm of root mean square deviation (Rq) as a function of logarithm of evaluation length (Rq-el) [13,14]. Datasets representing used and unused chert and obsidian flakes were measured with a laser profilometer. It was shown that Rq changed with evaluation length. Another study involving Rq-el indicated that chalk flint flakes used to cut pottery and wood, as measured by laser profilometry, could be successfully distinguished from the same surfaces prior to the use [15]. Additional research continued on chalk flint flakes used on shell, wood, dry hide, and soaked antler [16]. Relative length was used in [7] to discriminate used and unused tool regions. Area-scale analysis helped to distinguish unworn and worn regions of hide-cutting and wood-sawing obsidian flakes [17]. This was supported by applying statistical analysis—F-test versus scale. Other successful trials for other samples were described by Stemp et al. [18,19]. Area-scale fractal complexity has been shown to be a credible method that allows for the discrimination of loading of worn basalt flakes to cut oak branches [20]. Use-wear on experimental rhyolite stone flakes was discriminated by Álvarez et al. through calculation of fractal dimension [21,22]. Watson and Gleason applied area-scale analysis to determine the function and purpose of bone artifacts [23].

Considering all the above examples, research papers tend to focus on a single multiscale method applied to solve certain discriminating problem. Therefore, there is still a need to compare each method in terms of performance, i.e., how well they can help find functional correlations between formation process and resulting topography and its characterization parameters; between topography and their interaction with the environment; or how well they can facilitate discrimination between topographies. This study addresses the last problem by comparing different multiscale characterizations applied to discriminate topographies created by a combination of hot-rolling and mass-finishing. As a result of manufacturing processes, textures of apparent similarities and dissimilarities are obtained which are discernible at a certain scale or scales of observation.

## 2. Materials and Methods

### 2.1. Sample Preparations

**Figure 1:**
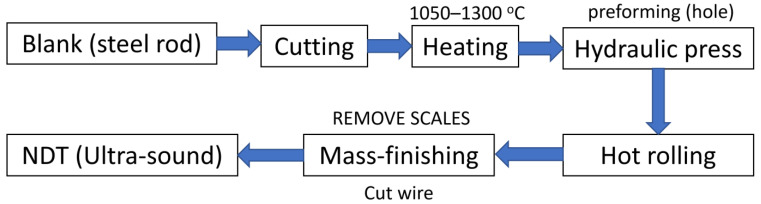
Diagram of technological process.

**Figure 2:**
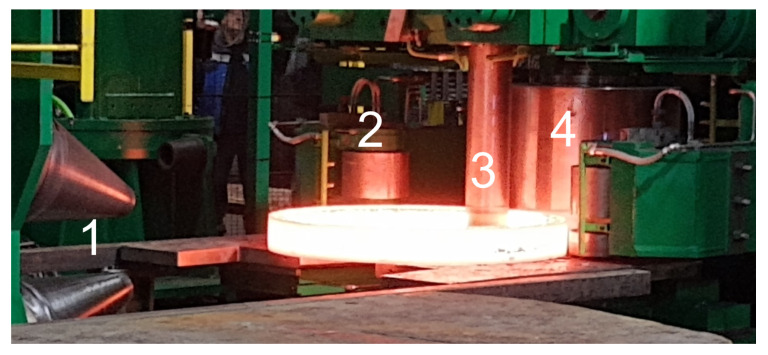
Ring during hot-rolling: 1—conical tools shaping upper and lower surfaces, 2—guiding roller, 3—internal roller and 4—external rollers.

**Figure 3:**
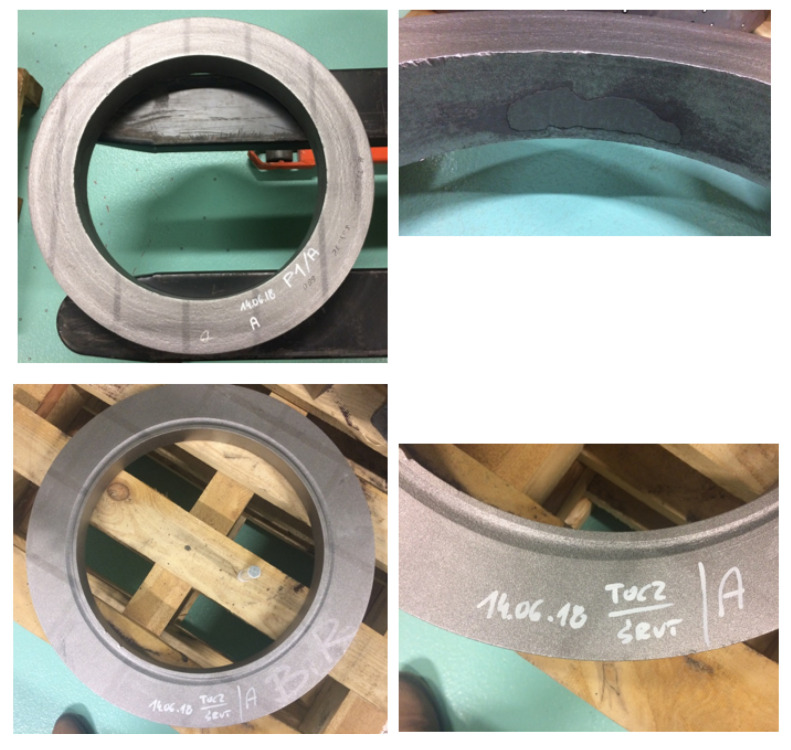
Ring R1 (**top row**) and R2 (**bottom row**); images in right column represent the magnified views of the corresponding rings.

Surfaces were created in a multistage process, which is schematically depicted in Figure 1. Firstly, a steel rod of 250 mm diameter was cut into 300 mm pieces, which were then heated in a furnace up to 1270 °C. Two materials were considered in this study: S355J2N (specimen A) and 42CrMo4T (specimen B). The next process was two-stage forging in which each piece was swollen, and a hole of 150 mm was cut centrally. The prepared blanks were then subjected to hot rolling. There were four surfaces shaped in this process by independent tools. Outer and inner cylindrical surfaces were formed by rollers, and two cones shaped the upper and lower faces (Figure 2). This allowed rings with a rectangular cross-sections to be obtained. Dimensions of both rings were as follows: specimen A ring—630 mm (outer diameter), 460 mm (inner diameter), and height 75 mm, with a weight of 86 kg, and specimen B—the outer diameter was equal to 620 mm, the internal diameter was 450 mm, and the height was 85 mm, with a weight of 96 kg (Figure 3). Formed parts were then mass-finished with spherical ceramic media (diameter between 0.8 and 1.25 mm) in order to remove scales, which was essential for achieving high fidelity of ultrasound testing. The medium feed rate was 0.5 and 1.1 m/min for rings A and B accordingly. The final product is a ring for a large size heavy-duty bearing. The surface topographies of each specimen and location (inner, outer, upper, and lower) were considered in this study.

### 2.2. Measurements

All surface were measured using a Hommel T8000 (JENOPTIK Industrial Metrology Germany GmbH, Villingen-Schwenningen, Germany) contact 3D profilometer equipped with a TKU 300/600 scanning head. A probe tip with a diamond needle end of 2 µm radius was used. Scanning speed was 0.15 mm/s. Measured regions were 4 mm by 4 mm. Sampling intervals differed in x (1 µm) and y (20 µm) directions. Four independent measurements were taken per every inner, outer, bottom, and upper representative location of each ring. For each measurement, form was removed using MountainsMap^®^ software (Digital Surf, Besançon, France).

### 2.3. Multiscale and Statistical Analysis

In this study, three different multiscale approaches were utilized. The first method was a series of band-pass filters together with calculation of surface texture parameters, as described in ISO/DIS 25178-2 and ISO 4287 for the measured datasets. MountainsMap software (version 8) was used to calculate all surface characterization parameters and to filter the topographies. Bandpass filtration was adopted from Berglund et al. [24] and was a combination of low-pass and high-pass Gaussian filters [25]. Firstly, a low-pass filter was applied using the upper wavelength cutoff, λ_uc_, followed by filtration with a high-pass filter at the lower wavelength cutoff, λ_lc_. The cutoffs refer to the wavelength where the filter has approximately 50% transmission. In this work, we used three different bandwidths, namely 20, 50, and 100 µm, which overlapped each other, which is similar to approach A from [26]. Lower and upper cutoff wavelengths are shown in Table 1. The number of bands depended on their widths and changed from 13 for the narrowest to 5 for the widest. The periodicity of the acquired topographies was evaluated with MountainsMap software to verify if the Gaussian filter could be applied in this study.

The other multiscale analysis was based on length-scale [7] and area-scale methods [27,28]. In the first one, the length of a profile is measured as a function of scale by a stepping exercise along the profile using what is essentially a virtual ruler. The length of the virtual ruler is the scale of measurement. Successive measurements are made at different scales using “rulers” of different lengths. For each measurement at a particular scale, the lengths of the virtual rulers are the same, and linear interpolations are used to locate the virtual steps between the sampling intervals in the profile. The relative length (Rel or RelL), as a function of scale, is determined by dividing the calculated (or measured) length by the nominal (or projected) length of the measured portion of the profile. The minimum possible value of Rel is 1 and is observed at the largest scales if the profile is level and long enough. At large scales, where the relative lengths are close to 1, the surface would be essentially smooth, whereas at a certain fine scale, called the smooth–rough crossover (SRC), the RelL has value sufficiently higher than 1. This translates to the observation that the texture can be regarded as rough below this scale. The extension from length of profiles to areas of irregular surfaces is conducted by the patchwork method. A measured surface (z = z(x,y)) is covered with triangular patches, in similarity to stepping a profile with line segments [7]. Both methods have been recently implemented in surface analysis software.

In the third method, we calculated components of curvature tensor in multiple scales by applying a 3D normal based method that is an evolution of the approach developed by Theisel et al. [29]. In order to estimate curvature tensor parameters at each scale in the analysis used here, height samples were taken from the original measurement. Down-sampling was used for approximating the appropriate corresponding scale. The point cloud, a regular array in x and y of measured height samples (z), was tiled to create triangular patches [30,31]. The statistical parameters (average and standard deviation) of maximal, minimal, mean, and Gaussian curvature were calculated as presented in [4]. Sixteen parameters were used in total: κ1a, κ1q, κ2a, κ1q, Ha_abs_, Hq_abs_, Ka_abs_, Kq_abs_, κ1a_abs_, κ1q_abs_, κ2a_abs_, κ1q_abs_, Ha_abs_, Hq_abs_, Ka_abs_, and Kq_abs_. Please note that the term “abs” in the subscript refers to the unsigned curvature. The full list of conventional profile and areal as well as length- and area-scale and curvature parameters used in this study is presented in Table A1 (Appendix A).

This study aims at comparing the performance of each multiscale method in the process of discriminating between measured samples. This is done based on characterization parameters, calculated using each multiscale methods, at each scale available in the measurement using two-way ANOVA. *p*-value is presented for each scale. The ability to discriminate surfaces with 95% or greater confidence was considered as sufficient (*p* < 0.05). Shapiro–Wilk tests were used to test for normal distributions of residuals.

## 3. Results and Analysis

### 3.1. Surface Topographies

**Figure 4:**
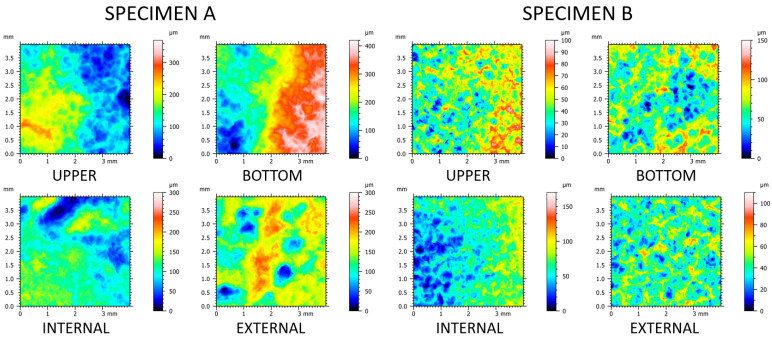
Renderings of topographic measurements capturing four different locations: upper, bottom, internal, and external of each of the two rings A and B.

Renderings of representative topographic measurements of four different locations for both rings are shown in Figure 4. Visual differences between those two specimen could be observed. Height scale was larger for specimen A (up to 410 µm) when compared to specimen B (170 µm utmost). Feature sizes appeared to follow the same tendency, as specimen B seemed to have more repetitive fine scale features: holes and hills. This could be quantified by using multiscale analysis. Topographies of specimen A did not possess apparent periodical features. The discrepancies in surface texture between rings might have been related to the different mass-finishing parameters and material properties. Topographic variety between locations at a single specimen might have been potentially caused by different tools used during hot-rolling. These differences were quantified using multiscale analysis. The average periodicity was low and equal to 12.14% (SD = 3.32%) with two outliers of maximum 21.83% and 22.15%. We considered those results supportive to the use of the Gaussian bandpass filter in this study.

### 3.2. Bandpass Filter

**Figure 5:**
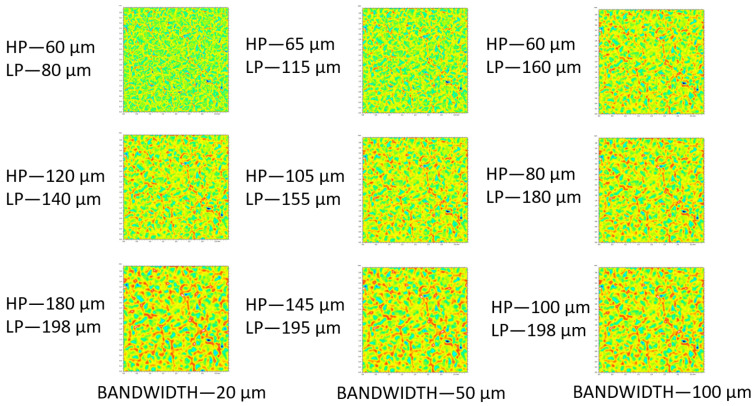
Renderings of bandpass filtered topographies at the upper location of Specimen A using three different bandwidths: 20, 50, and 100 µm. Please note that HP and LP stand for cut-off wavelengths of high- and low-pass filters, respectively.

**Figure 6:**
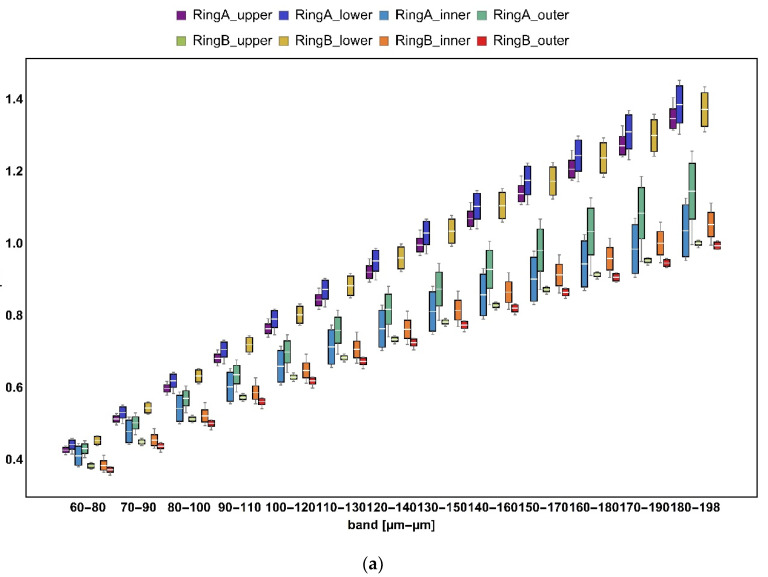

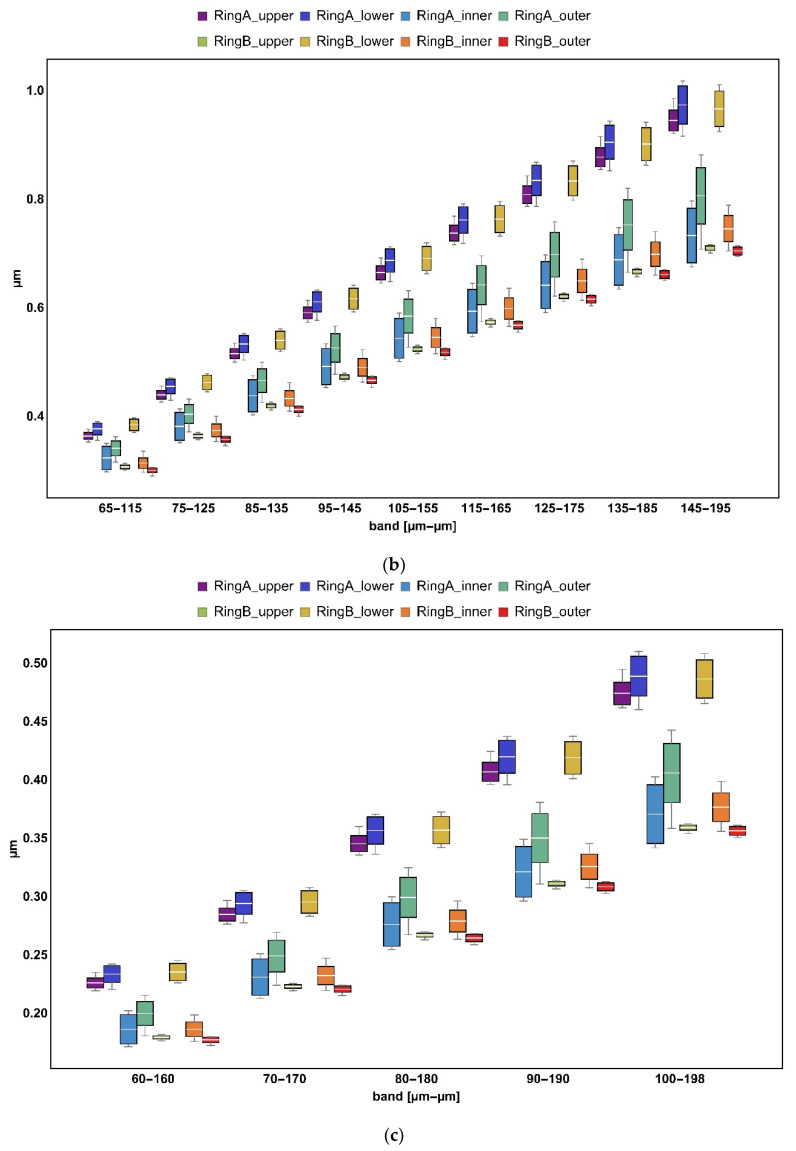
Evolution of arithmetic mean height (Sa) with scale for three different bandwidths: (**a**) 20, (**b**) 50, and (**c**) 100 µm.

**Figure 7:**
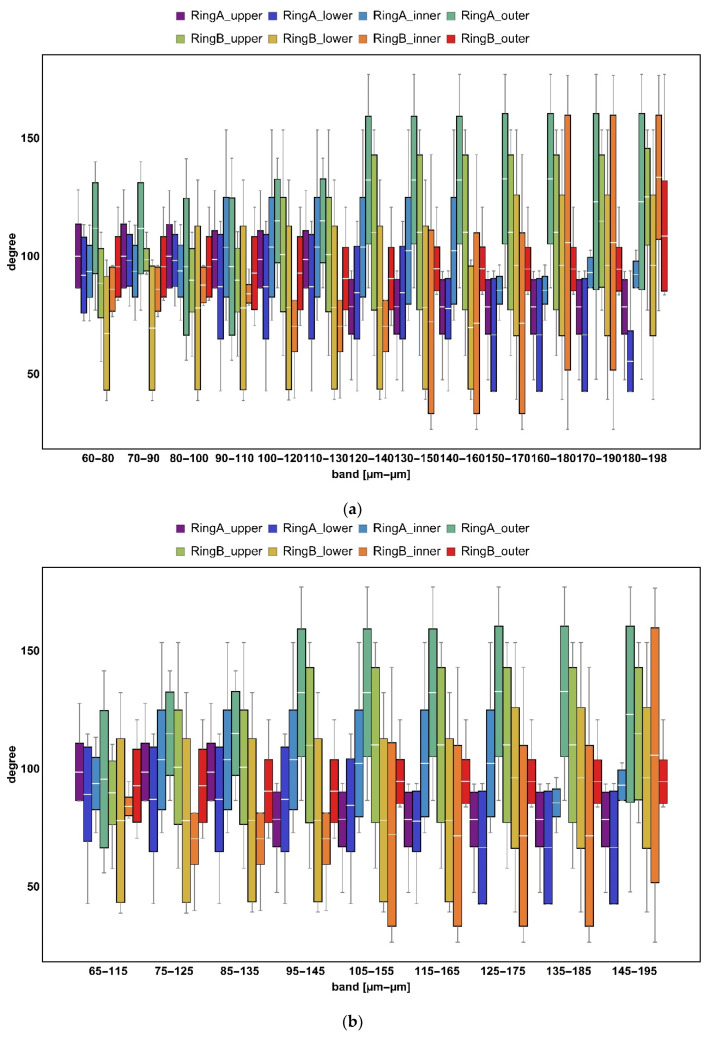

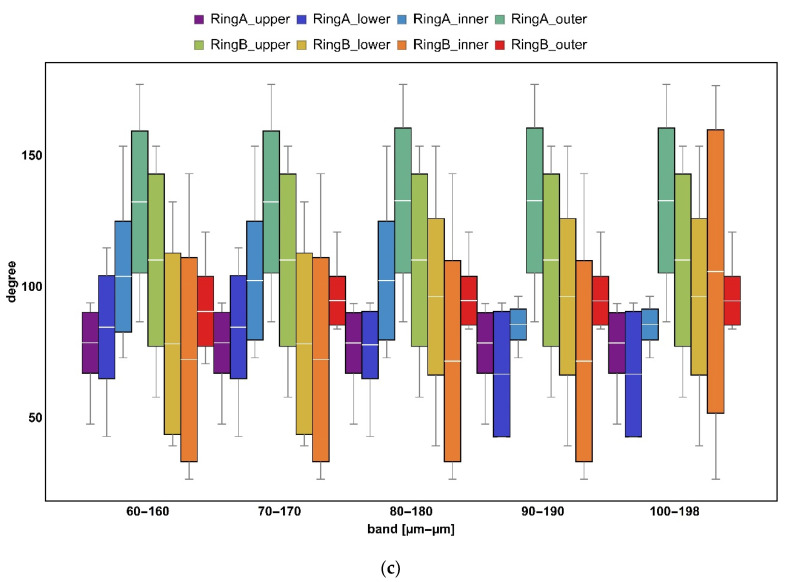
Evolution of texture direction (Std) with scale for three different bandwidths: (**a**) 20, (**b**) 50, and (**c**) 100 µm.

Results of bandpass filtration with three different bandwidths—20, 50, and 100 µm—of an exemplary upper surface of specimen A are depicted in Figure 5. The effect of separation of topographic features was the most evident for the narrowest bandwidth. The topographies filtered with the widest bandwidth seemed to differ the least from each other, as they contained a larger set of wavelengths that overlapped with others. This can be supported by presenting the evolution of roughness parameters with bands as a function of central wavelength. This kind of plot can be presented in linear, log-linear, or log-log scales [1,32]. In this paper, we visually demonstrated only a selected range of characterization parameters, which, by definition, describe the analyzed surface morphologies. This included arithmetic mean height (Sa) (Figure 6) and Std (Figure 7). Other calculated ISO standard parameters as a function of band are available in the supplementary materials. Rt and also Ra tended to increase as the band slid to higher wavelengths. In most engineering surfaces, when decomposed using Fourier transforms, the amplitude of the longest wavelengths is often the highest [1]. This translates to the trend of observed height amplitude parameters (Ra, Rt, Rz, Rv, and Rq for profiles as well their respective areal analogs Sa, Sz, Sv, and Sq), the values of which grow with band progression. For each band, no matter its width, two groups were visually distinctive, namely the upper surface of Ring A, the lower surfaces of both Ring A and B, and the other five surfaces. By analyzing the obtained images, it was found that the application of the mass-finishing treatment and the level of removal of the layer of surface contamination of the workpiece exposed the original material and its texture. In addition, a strong abrasive stream affected the asperities of the primary surface, forming it in a more isotropic way. By using bandpass filters, the share of a given wavelength component was limited or enhanced, and thus the functional parameters described mainly roughness. The use of filtration for the 100 µm band was characterized by more distinct valleys and softer mapping of hills. However, a 20 µm bandwidth filter showed an impact for slight changes in structure on the surface represented, in particular those related to hills. This effect was especially important when using multiscale analysis to quantify the surface condition. Different manufacturing effects could not be noted for Std. This parameter described the dominant texture direction by indicating its angle with respect to the horizontal axis (see Figure 7). For the analyzed datasets, it was found to be the least scale dependent. This suggests that any residual lay potentially resulting from hot rolling, which by definition was directional, was removed during mass-finishing, making the surfaces isotropic. As far as hybrid parameters are concerned, attention was drawn to interesting relationships related to the width of the filtration bandwidth. This effect was clearly visible, for example, for the Sdq parameter for a bandwidth of 20 µm. It should be noted that for the surfaces on ring A, there was a monotonic increase in the value of this parameter, while for isotropic surfaces (ring B) its value stabilized already at the filtration defined by the following wavelengths: LP = 110 µm/HP = 130 µm. In addition, this parameter was less sensitive to the filter bandwidth above the specified limit. The parameters from the volume and feature group showed a tendency to be monotonic as a function of the filter bandwidth and wavelength.

### 3.3. Length- and Area-Scale Analysis

**Figure 8:**
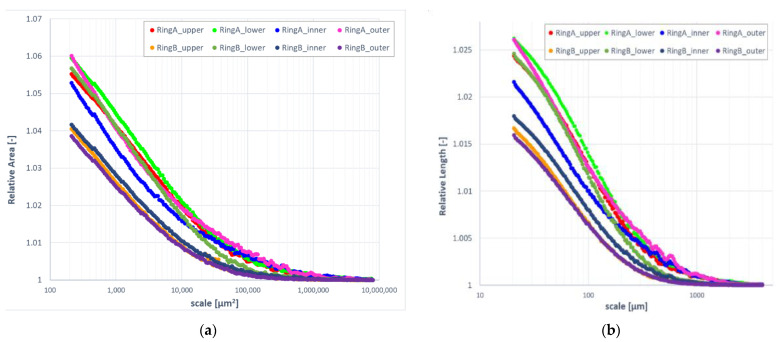
Evolution of relative area (**a**) and length (**b**) as a function of scale.

**Figure 9:**
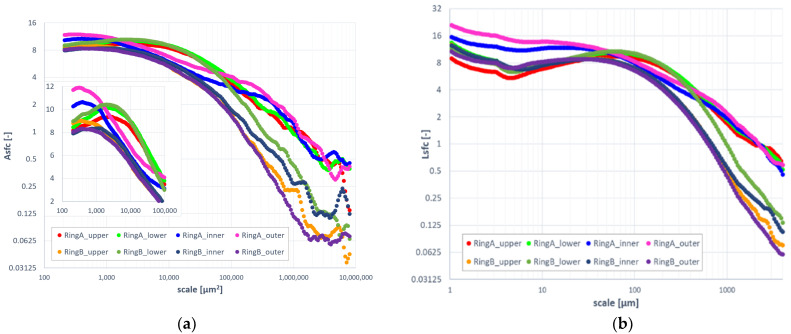
Evolution of area-scale (**a**) and length-scale (**b**) fractal complexity as a function of scale.

Evolution of relative area and length as a function of scale indicated differences between surfaces formed on ring A and B (Figure 8). At finer scales (<15,000 μm^2^ for area and <150 μm for length), upper, inner, and outer regions of specimen B took evidently smaller values of the geometric measures when compared to the others. The lower surface of the same ring appeared to be similar to the lower surface of ring A, when considering the same range of scales. At larger scales, the results for both rings could be clearly differentiated. This became even clearer when considering complexity (Figure 9). Surface topographies measured on specimen B exhibited noticeably lower fractal complexity than their counterparts. This might indicate that second-stage processing via mass-finishing plays a dominant role in the formation of distinctive surface topographies. Differences between surfaces expressed through area- and length-scale analysis of the corresponding location on both rings were rather subtle and hard to be visually detected based on the figures.

### 3.4. Curvature

**Figure 10:**
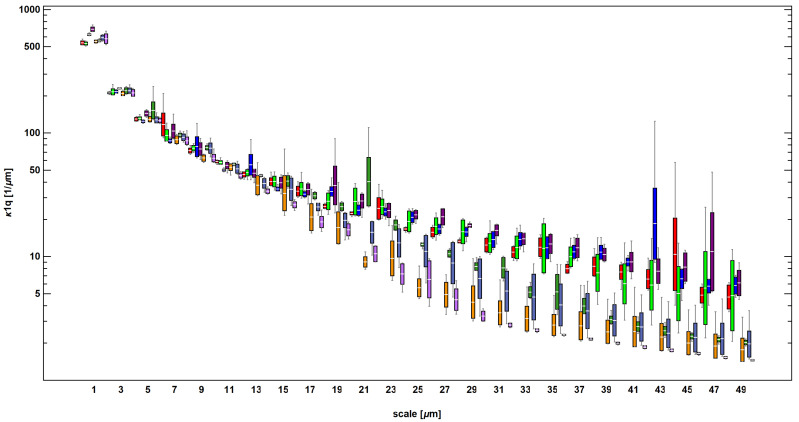
Evolution of standard deviation of maximum curvature (κ1q) as a function of scale.

For most curvature parameters, evident differences between the two rings became visible at scales starting from 27 µm. Exemplary results showing the evolution of κ1q as a function of scale are depicted in Figure 10. At very fine scales, the shape of the surface topography quantified by the curvature appeared to be similar. This might suggest that texture formation at the microscale, which is a product of two-stage processing, results in the similar surface morphology. This observation was true for all parameters apart from average parameters of signed curvature (maximum, minimum, mean, and Gaussian). This means that variability of surface curvature as expressed by standard deviation or mean deviation from flat surface (average parameters of unsigned curvature quantify how the surface shape differs from curvature equal to zero) appears to be an appropriate characterization parameter for discrimination. Visually, no clear trends could be used to analyze the effect of first stage processing through hot-rolling (between corresponding regions of both specimen). This corresponded to the same observations for length- and area-scale analysis, where evident distinctions between each ring could be noted at larger scales. Further illustrations showing the evolutions of other curvature parameters are shown in the supplementary materials to this study.

### 3.5. Discrimination Analysis

Using two-way ANOVA, height parameters are generally appropriate characterizations, which can indicate statistically if the results are different when considering hot rolling and mass-finishing individually or as their product. For all three bands, *p*-values for Sa and Sq were lower than 0.05. Skewness and kurtosis failed to be used as a statistical discriminator against 2nd stage processing for bandwidth equal to 50 µm. Other height parameters did not generally provide sufficient confidence levels in the analyzed case. Hybrid and volume groups exhibited strong potential for differentiation between surface topographies considering all three factors with an exception for Vm (material volume) and Vmp (peak material volume) for the narrowest and shortest bandwidth (60–80 µm) when considering both factors as a superposition. This corresponded well to the visual observation noted for geometric characterization parameters, which allowed significant enough discrimination at the finest scales. Most feature parameters could be used to differentiate surfaces taking into account only the location on each ring. Surface texture ratio (Str) could be utilized as a sole spatial parameter to discriminate against both factors, and individually only for narrow ranges of scales. Similar observations could be made for profile parameters evaluated for corresponding groups.

**Figure 11:**
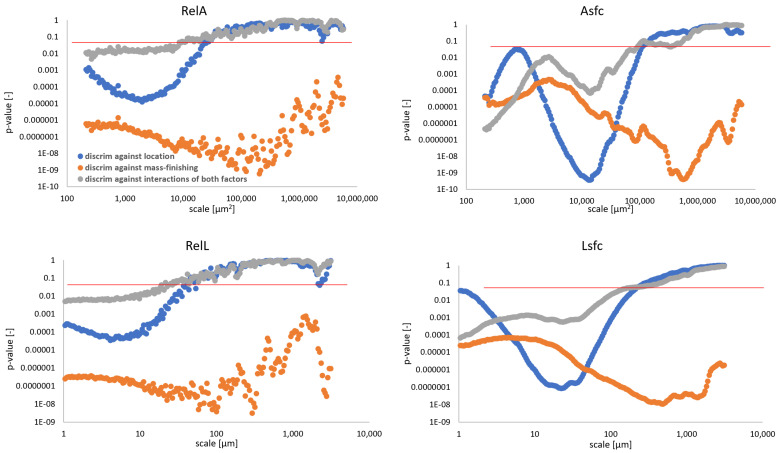
Evolution of *p*-value calculated in the discrimination analysis of two independent formation factors and their product as a function of scale for area- and length-scale parameters: RelA (**top left**), Asfc (**top right**), RelL (**bottom left**), Lsfc (**bottom right**). Please note that red line indicates *p* = 0.05 at each graph.

Fractal complexity exhibited superior performance in discrimination against all factors and their combinations when compared to relative length and area (Figure 11). The latter parameters could be used only when finer scales were considered. Discrimination against mass-finishing was confident no matter the parameter. This confirms the visual observations as presented in the previous sections. Asfc allowed confident differentiation between factors for fine and medium scales (<10,000 μm^2^). The same phenomenon was noted for Lsfc and scales up to 150 μm.

**Figure 12:**
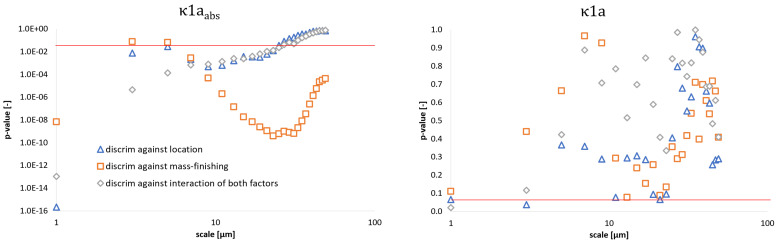
Evolution of *p*-value calculated in the discrimination analysis of two independent formation factors and their product as a function of scale for average absolute maximum curvature (**left**) and their signed counterpart (**right**). Please note that the red line indicates *p* = 0.05 at each graph.

Curvature could also be used a discriminant only when considering measures of variability (Figure 12). For average maximum curvature (κ1a and Ha), *p*-values generally exceeded 0.05 for most of scales. Average minimum and Gaussian curvature performed better for all factors at scales <13 μm. Unsigned curvature, which did not focus on differentiation between convex and concave regions but quantified the magnitude of local surface bending, provided significantly confident discrimination for scales up to 25 μm. Contrary to length- and area scale complexity, curvature failed to detect statistically significant differences between surfaces for the largest analyzed scales, apart from average minimum curvature (κ2a), as presented in the supplementary materials.

## 4. Discussion

The ability to discriminate between surfaces is essential for in-depth understanding of how topographies should be designed to enhance their performance or optimized in terms of their fabrication. Discrimination analysis is the important first step in these understandings, as surfaces which cannot be confidently differentiated usually do not perform well when searching for correlations with the functional behavior or manufacturing parameters. Multiscale analysis can help in identifying which surface characterization parameter and at what scales is the most significant for telling surfaces apart.

In this study, we showed that all four studied multiscale methods performed well in discriminating two-stage processed surfaces. For the bandwidth method, the simplest and most commonly used height parameters showed good results for all three bandwidths. These parameters are most sensitive to the longest wavelengths [33] and characterize how, on average, the topography is rough, which appeared to be enough for differentiating between the two stages of processing.

Topographic features of certain shape(s) and dimensions are usually signatures of a particular formation process. Milling, turning, or rolling typically create directional marks, while selective laser sintering leads to the formation of mosaics of directional wrinkles, holes, and not fully melted powder conglomerates [34]. These features are best discernible at particular scale(s) of observation and may manifest themselves differently when observing across different scales. Discrimination considering narrow scales and using appropriate geometric characterization parameters becomes essential in better understanding the nature of manufacturing processes and its control [35]. In our study, ISO standard parameters, which describe the geometric properties of surface topographies, generally performed well when only a narrow range of scales was considered. Similar observations could be made for curvature, length-, and area-scale parameters, which also generally failed to tell surfaces apart at the largest scales.

The height amplitude of surface topography is significantly different between the two rings (roughly twice when comparing sample A with B). This was caused by different processing and material properties of the two rings. The difference in height amplitude is reflected in height parameters (both profile and areal), and relative length and area and their derivatives. Curvature analysis revealed that the shape of the topographic features, as quantified with the curvature statistical parameters, is similar at the finest scales (≤17 µm) and cannot be discriminated against mass finishing at this range of scales. This might mean that the microgeometry of the samples is affected in the same manner no matter the second stage process, while the manufacturing and material are factors when considering shape characteristics of large scale features.

There is an abundance of multiscale methods [1], but they are most often used as a single tool aimed at describing particular topography-related effect. Recently, new studies have emerged that discuss the differences in the performance of each method. Guilbert et al. focused on three segmentation techniques, namely patchwork, box, and motifs multiscale decomposition, to study PEEK polymer surfaces subjected to various abrasive processes [36]. The authors found that all the methods successfully detected crossover scale, separating larger and smaller abrasion regions. In terms of discrimination, those three techniques complemented each other. In another work, Serafin et al. investigated length-scale and profile curvature of pure iron in terms of their oxidation performance [37]. They found that both methods are useful for discrimination of differently treated samples at the scale close to the scale of ions that take part in a chemical reaction of oxidation. Relating to curvature only and its ability to confidently tell surfaces apart, Maleki et al. showed that the hereby presented 3D curvature method and the Bigerelle–Nowicki approach exhibited the best performance for computer-generated fractal surfaces, sine waves, and real engineering examples [38]. In [32], the authors found that motifs and curvature differentiate EDMed textures well when analyzing scales associated with the size of the most typical features of their morphology, i.e., craters, which were formed as a result of electric discharges. All the aforementioned studies did not conclude that there is a one single universal multiscale method that can be applied successfully in any case. Our study also proved that statement, as most of the hereby studied methods indicated that looking at the finest scales allows the most confident differentiation. This was also intuitively noted by visual inspection of the measured surface topographies.

From a practical perspective, the most favorable tools in surface metrology are the ones which are the simplest to understand and easiest to use. For the studied case, basic ISO standard parameters Sa and Sq exhibited superior performance in discriminating between both factors and their combination for all three analyzed bandwidths. Bandpass filtering is also easy to comprehend and widely applied in computational software. Although that method inherently lacks the insightfulness of the other multiscale techniques, its aforementioned advantages cannot be neglected.

The limitation of this study is mostly based on the applied measurement technique (3D profilometry), which resulted in differences in x- and y-sampling intervals. The larger sampling interval in the latter direction (between measured profiles) set constraints in the shortest possible cut-off wavelengths and, as a consequence, reduced the number bands. It also had an effect in area-scale analysis and curvature estimation, as they both involve tiling the measured surface at some step of the calculation procedure. Considering the evolution of curvature parameters with scale, the evident distinction between results for Ring A and B can be discerned for scales starting from circa 20 µm, which is equal to sampling intervals in the y-direction. Nonetheless, the discrimination versus both factors and their combination was possible with any of the studied method. The effect of sampling technique and a detailed analysis of the mechanisms that stood behind the obtained results for each of the methods deserve a separate study with, perhaps, low- or non-periodic artificially generated datasets of deeply well-known characteristics.

The hereby presented study focused on two types of multiscale analysis: bandpass and geometric (curvature, length- and area-scale). Other studies concentrate on methods within one [36,37,38] or two groups [33]. To fully study the performance of multiscale methods, more effort is needed to verify other techniques applied for discrimination of surfaces of various morphologies. In the future, this can lead to the creation of a multiscale analysis framework that can assist potential users in the challenges they face while characterizing the complexity of topographies they study. This paper, among others, is a step ahead in this journey.

## 5. Conclusions

In this study, we evaluated the discrimination performance of four multiscale methods, namely bandpass filtration, length- and area-scale analysis, as well as multiscale curvature analysis, applied to a set of topographies from two-staged processed steel rings, measured at four different locations with 3D contact profilometer. The ability to tell the surfaces apart was quantified using two-way ANOVA and calculating *p*-value, considering mass-finishing and location as two independent factors. The conclusions of this study can be summarized as follows:

- All four studied multiscale methods performed generally well in discriminating against each factor and their combinations;
- Bandpass filtration using Sa and Sq exhibited the best performance as *p*-value < 0.05 for all three bands. Skewness and kurtosis failed to be used as a statistical discriminator against mass finishing for a bandwidth equal to 50 µm. Other height parameters did not generally provide sufficient confidence levels in the studied case. Hybrid and volume group parameters performed well at differentiation between surface topographies considering all factors with the exceptions of Vm and Vmp for the narrowest and shortest bandwidth (60–80 µm) when considering both factors as superpositions. Most feature parameters can be used to discriminate surfaces taking into account only the location on each ring. Spatial parameters were found to perform poorly. Similar conclusions can be drawn for profile characterization counterparts;
- Asfc and Lsfc both exhibited superior performance in discrimination against all factors and their combinations when compared to RelL and RelA. The latter parameters could be used only when finer scales were considered. Discrimination against mass-finishing was confident no matter the parameter derived from those methods;
- Curvature can be used as a discriminant only when considering measures of variability. Unsigned curvature provides significantly confident discrimination for finer scales.

## Figures and Tables

**Table 1:** Lower and upper cutoff and center wavelengths used in the bandpass filtering method.

Bandwidth = 20 μm			Bandwidth = 50 μm			Bandwidth = 100 μm		
λ_lc_ (μm)	λ_center_ (μm)	λ_uc_ (μm)	λ_lc_ (μm)	λ_center_ (μm)	λ_uc_ (μm)	λ_lc_ (μm)	λ_center_ (μm)	λ_uc_ (μm)
60	70	80	65	90	115	60	110	160
70	80	90	75	100	125	70	120	170
80	90	100	66	110	135	80	130	180
90	100	110	95	120	145	90	140	190
100	110	120	105	130	155	100	150	198
110	120	130	115	140	165	–	–	–
120	130	140	125	150	175	–	–	–
130	140	150	135	160	185	–	–	–
140	150	160	145	170	195	–	–	–
150	160	170	–	–	–	–	–	–
160	170	180	–	–	–	–	–	–
170	180	190	–	–	–	–	–	–
180	190	198	–	–	–	–	–	–

## Data Availability

Data is contained within the article or supplementary material.

## Supplementary Materials

The following are available online at https://www.mdpi.com/article/10.3390/ma14227044/s1. The additional material include the detailed computations of all ISO standard parameters, each presented as a box-and-whisker plot and as a function of band. Three bandwidths are available: 20, 50, and 100 µm. The materials also contain the evolution of all considered curvature parameters, each also presented as a function of scale and in the form of a box-and-whisker plot. Rose plots and height distributions for representative bandpass filtered surfaces are also included, together with the calculations of isotropy and periodicity for unfiltered datasets.

## Appendix A

**Table A1:** Abbreviations of standard surface characterization parameters and their full meanings.

Abbreviation	Full Name
Rp	Maximum peak height of the roughness profile
Rv	Maximum valley depth of the roughness profile
Rz	Maximum height of roughness profile
Rc	Mean height of the roughness profile elements
Rt	Total height of roughness profile
Ra	Arithmetic mean deviation of the roughness profile
Rq	Root-mean-square (RMS) deviation of the roughness profile
Rsk	Skewness of the roughness profile
Rku	Kurtosis of the roughness profile
Sq	Root-mean-square height
Ssk	Skewness
Sku	Kurtosis
Sp	Maximum peak height
Sv	Maximum pit height
Sz	Maximum height
Sa	Arithmetic mean height
Smr	Areal material ratio
Smc	Inverse areal material ratio
Sxp	Extreme peak height
Sal	Autocorrelation length
Str	Texture-aspect ratio
Std	Texture direction
Sdq	Root-mean-square gradient
Sdr	Developed interfacial area ratio
Vm	Material volume
Vv	Void volume
Vmp	Peak material volume
Vmc	Core material volume
Vvc	Core void volume
Vvv	Pit void volume
Spd	Density of peak
Spc	Arithmetic mean peak curvature
S10z	Ten point height
S5p	Five point peak height
S5v	Five point pit height
Sda	Mean dale area
Sha	Mean hill area
Sdv	Mean dale volume
Shv	Mean hill volume
Sku	Core roughness depth
Spk	Reduced summit height
Svk	Reduced valley depth
Smr1	Upper bearing area
Smr2	Lower bearing area
RelL	Relative length
RelA	Relative area
Lsfc	Length-scale fractal complexity
Asfc	Area-scale fractal complexity
κ1a	Average maximum curvature
κ1q	Standard deviation of maximum curvature
κ2a	Average minimum curvature
κ2q	Standard deviation of minimum curvature
Ha	Average mean curvature
Hq	Standard deviation of mean curvature
Ka	Average Gaussian curvature
Kq	Standard deviation of Gaussian curvature
κ1a_abs_	Average absolute maximum curvature
κ1q_abs_	Standard deviation of absolute maximum curvature
κ2a_abs_	Average absolute minimum curvature
κ2q_abs_	Standard deviation of absolute minimum curvature
Ha_abs_	Average absolute mean curvature
Hq_abs_	Standard deviation of absolute mean curvature
Ka_abs_	Average absolute Gaussian curvature
Kq_abs_	Standard deviation of absolute Gaussian curvature

## References

[1] Brown C.A., Hansen H.N., Jiang X.J., Blateyron F., Berglund J., Senin N., Bartkowiak T., Dixon B., Le Goïc G., Quinsat Y. (2018). Multiscale analyses and characterizations of surface topographies. CIRP Ann..

[2] Pawlus P., Wieczorowski M., Mathia T. (2014). The Errors of Stylus Methods in Surface Topography Measurements.

[3] ISO 25178-1 Geometrical Product Specifications (GPS)—Surface Texture: Areal—Part1. https://www.iso.org/standard/46065.html.

[4] Peta K., Bartkowiak T., Gałek P., Mendak M. (2021). Contact angle analysis of surface topographies created by electric discharge machining. Tribol. Int..

[5] Bigerelle M., Mazeran P.E., Gong W., Giljean S., Anselme K. (2011). A Method to determine the Spatial Scale Implicated in Adhesion. Application on Human Cell Adhesion on Fractal Isotropic Rough Surfaces. J. Adhes..

[6] Jordan S.E., Brown C.A. (2006). Comparing Texture Characterization Parameters on Their Ability to Differentiate Ground Polyethylene Ski Bases. Wear.

[7] American Society of Mechanical Engineers (2009). ASME Standard, B46.1; Surface Texture, Surface Roughness, Waviness and Lay.

[8] ISO 25178-2 Geometrical Product Specifications (GPS))—Surface Texture: Areal—Part 2. https://www.iso.org/standard/42785.html.

[9] Brown C.A., Etievant D., Shao Y., Nevers S., Bartkowiak T. Multiscale Outlier Filtering with Curvature and Higher Spatial Finite Differences. Proceedings of the 16th International Conference on Metrology and Properties of Engineering Surfaces.

[10] Brown C.A., Etievan T.D., Shao Y., Nevers S., Bartkowiak T. (2017). Measurement Equipment with Outlier Filter. U.S. Patent.

[11] Ulcickas V. (2000). Using Area-scale Relations to Investigate Measured Fracture Toughness in Y-TZP Materials. Ceramic Trans..

[12] Mezghani S., El Mansori M., Massaq A., Ghidossi P. (2008). Correlation Between Surface Topography and Tribological Mechanisms of the Belt-finishing Process Using Multiscale Finishing Process Signature. Comptes Rendus Méc..

[13] Stemp W.J. (2014). A Review of Quantification of Lithic Use-wear Using Laser Profilometry: A Method Based on Metrology and Fractal Analysis. J. Archaeol. Sci..

[14] Stemp W.J., Stemp M. (2001). UBM Laser Profilometry and Lithic Use-wear Analysis: A Variable Length Scale Investigation of Surface Topography. J. Archaeol. Sci..

[15] Stemp W.J., Stemp M. (2003). Documenting Stages of Polish Development on Experimental Stone Tools: Surface Characterization by Fractal Geometry Using UBM Laser Profilometry. J. Archaeol. Sci..

[16] Stemp W.J., Childs B.E., Vionnet S., Brown C.A. (2009). Quantification and Discrimination of Lithic Use-wear: Surface Profile Measurements and Length-scale Fractal analysis. Archaeon.

[17] Stemp W.J., Chung S. (2011). Discrimination of Surface Wear on Obsidian Tools Using LSCM and RelA: Pilot Study Results. Scanning.

[18] Stemp W.J., Andruskiewicz M.D., Gleason M.A., Rashid Y.H. (2015). Experiments in Ancient Maya Blood-Letting: Quantification of Surface Wear on Obsidian Blades. Archaeol. Anthropol. Sci..

[19] Stemp W.J., Lerner H.J., Kristant E.H. (2013). Quantifying Microwear on Experimental Mistassini Quartzite Scrapers: Preliminary Results of Exploratory Research Using LSCM and Scale-sensitive Fractal Analysis. Scanning.

[20] Key A., Stemp W.J., Morozov M., Proffitt T., Torre I. (2015). Is Loading a Significantly Influential Factor in the Development of Lithic Microwear. An Experimental Test Using LSCM on Basalt from Olduvai Gorge. J. Archaeol. Method Theory.

[21] Álvarez M., Fuentes N.O., Favret E.A., Dolce M., Forlano A. (2012). Quanti-fying Use-wear Traces through RIMAPS and Variogram Analyses. Archaeol. Anthropol. Sci..

[22] Favret E.A., Fuentes N.O., Álvarez M.R. (2004). RIMAPS and Variogram Analyses of Microwear Traces in Experiments with Stone Tools. Microsc. Micro Anal..

[23] Watson A.S., Gleason M.A. (2016). A Comparative Assessment of Texture Analysis Techniques Applied to Bone Tool Use-Wear; Surface Topography. Metrol. Prop..

[24] Berglund J., Agunwamba C., Powers B., Brown C.A., Rosén B.G. (2010). On discovering relevant scales in surface roughness measurement an evaluation of a band-pass method. Scanning.

[25] Muralikrishnan B., Raja J. (2009). Computational Surface and Roundness Metrology.

[26] Berglund J., Wiklund D., Rosén B.G. (2011). A method for visualization of surface texture anisotropy in different scales of observation. Scanning.

[27] Brown C.A., Charles P.D., Johnsen W.A., Chesters S. (1993). Fractal Analysis of Topographic Data by the Patchwork Method. Wear.

[28] Brown C.A., Johnsen W.A., Charles P.D. (1994). Method of Quantifying the Topographic Structure of a Surface. U.S. Patent.

[29] Theisel H., Rössl C., Zayer R., Seidel H.P. Normal based estimation of the curvature tensor for triangular meshes. Proceedings of the 12th Pacific Conference on Computer Graphics and Applications.

[30] Bartkowiak T., Brown C.A. (2018). A characterization of process-surface texture interactions in micro-electrical discharge machining using multiscale curvature tensor analysis; Journal of Manufacturing Science and Engineering. Trans. ASME.

[31] Bartkowiak T., Brown C.A. (2019). Multiscale 3D curvature analysis of processed surface textures of aluminum alloy 6061 T6. Materials.

[32] Bigerelle M.M., Mathia T., Bouvier S. (2012). The multi-scale roughness analyses and modeling of abrasion with the grit size effect on ground surfaces. Wear.

[33] Bartkowiak T., Mendak M., Mrozek K., Wieczorowski M. (2020). Analysis of Surface Microgeometry Created by Electric Discharge Machining. Materials.

[34] Bartkowiak T., Berglund J., Brown C.A. (2020). Multiscale Characterizations of Surface Anisotropies. Materials.

[35] Braatz R.D., Alkire R.C., Seebauer E., Rusli E., Gunawan R., Drews T.O., Li X., He Y. (2006). Perspectives on the Design and Control of Multiscale Systems. J. Process Control.

[36] Guibert R., Hanafi S., Deltombe R., Bigerelle M., Brown C.A. (2020). Comparison of three multiscale methods for topographic analyses. Surf. Topogr. Metrol. Prop..

[37] Serafin D., Bartkowiak T., Nowak W.J., Wierzba B. (2020). Influence of microgeometry of iron surface on the oxidation process)—A comparison of multiscale geometric methods and their applicability. Appl. Surf. Sci..

[38] Maleki I., Wolski M., Woloszynski T., Podsiadlo P., Stachowiak G. (2019). A Comparison of Multiscale Surface Curvature Characterization Methods for Tribological Surfaces. Tribol. Online.

