# Supplementary material for: Discrimination of Surface Topographies Created by Two-Stage Process by Means of Multiscale Analysis
Source: Materials (Basel). 2021 Nov 20;14(22):7044. doi: 10.3390/ma14227044 (PMC8624860; doi:10.3390/ma14227044)
Supplement: Supplementary file 1 [file materials-14-07044-s001.zip › supplementary materials/rose plots and heights distribution/RingA_inner_1 100-198.pdf]

Identity card

Name:	P1A_wewnetrzna_1 100-198 > P1A_wewnetrzna_1 100-198 > P1A_wewnetrzna_1 100-198 > P1A_wewnetrzna_1 100-198

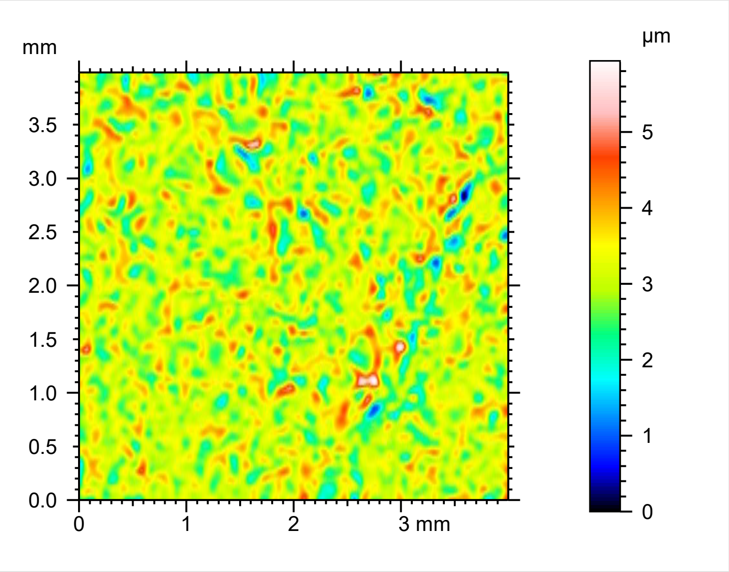

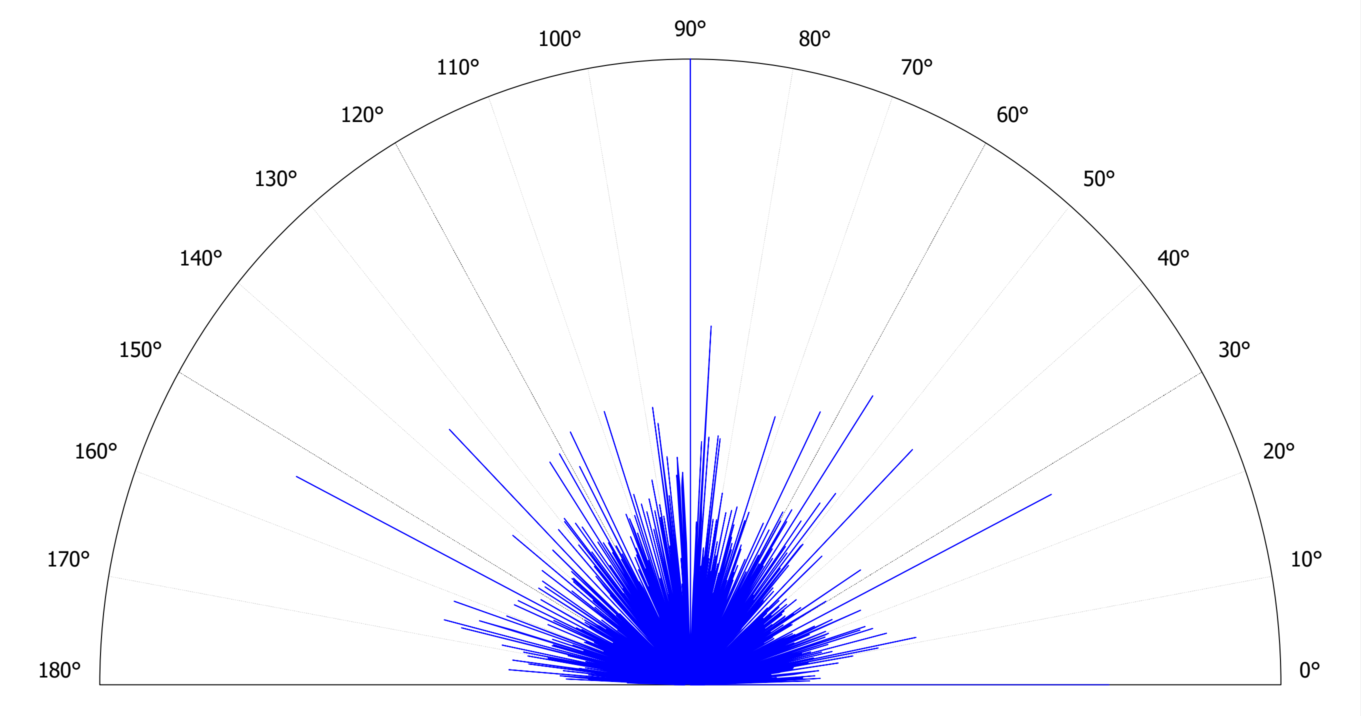

Parameters	Value	Unit
Isotropy	78.0	%
First Direction	90.0	°
Second Direction	154	°
Third Direction	0.107	°

MountainsMap® Premium 7.4.8083

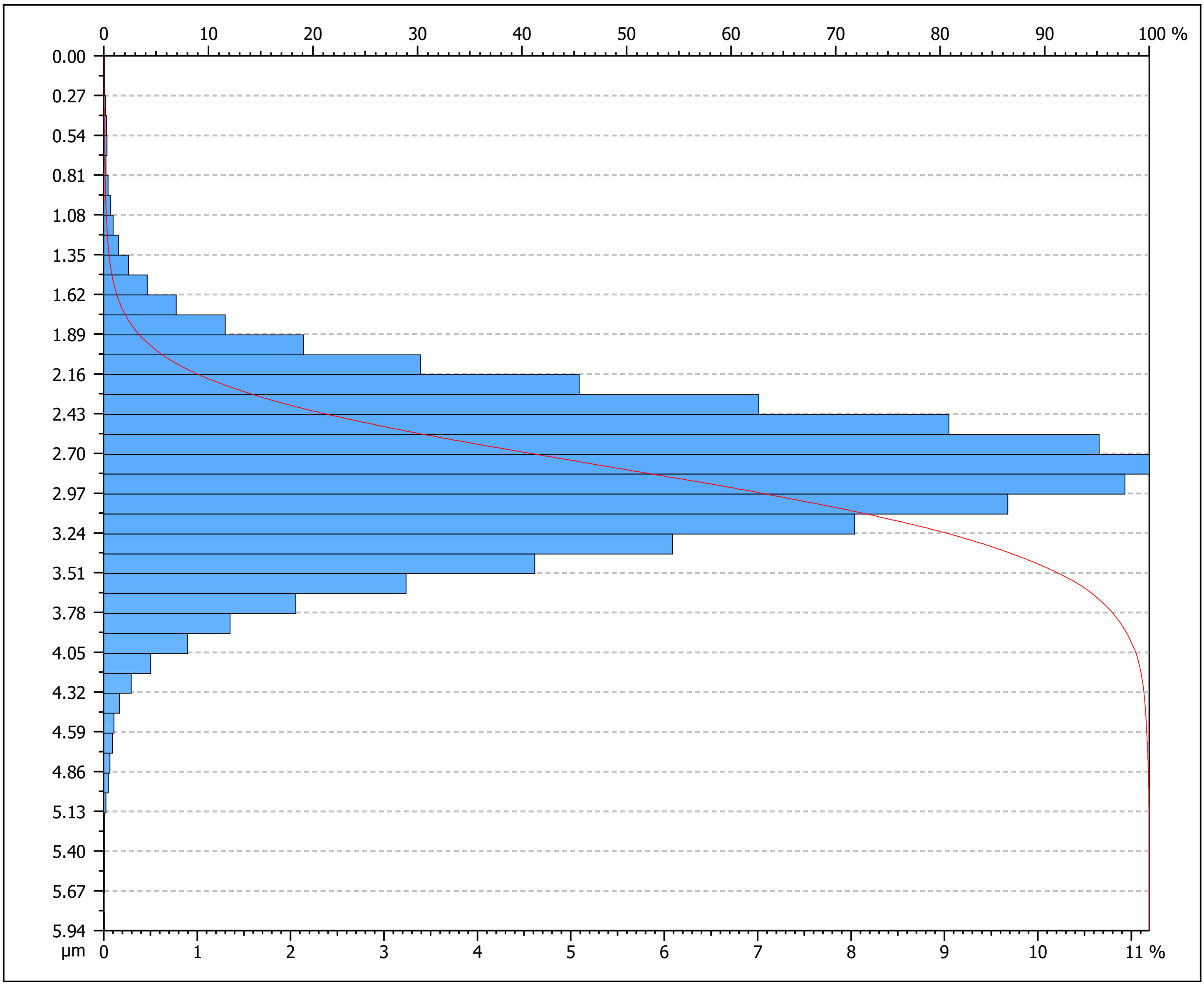
0 10 20 30 40 50 60 70 80 90 100 %
0.00
0.27
0.54
0.81
1.08
1.35
1.62
1.89
2.16
2.43
2.70
2.97
3.24
3.51
3.78
4.05
4.32
4.59
4.86
5.13
5.40
5.67
5.94
µm
0 1 2 3 4 5 6 7 8 9 10 11 %
