# Supplementary material for: Discrimination of Surface Topographies Created by Two-Stage Process by Means of Multiscale Analysis
Source: Materials (Basel). 2021 Nov 20;14(22):7044. doi: 10.3390/ma14227044 (PMC8624860; doi:10.3390/ma14227044)
Supplement: Supplementary file 1 [file materials-14-07044-s001.zip › supplementary materials/rose plots and heights distribution/RingA_inner_1 120-140.pdf]

**Identity card**	
Name:	P1A_wewnetrzna_1 120-140 > P1A_wewnetrzna_1 120-140 > P1A_wewnetrzna_1 120-140 > P1A_wewnetrzna_1 120-140

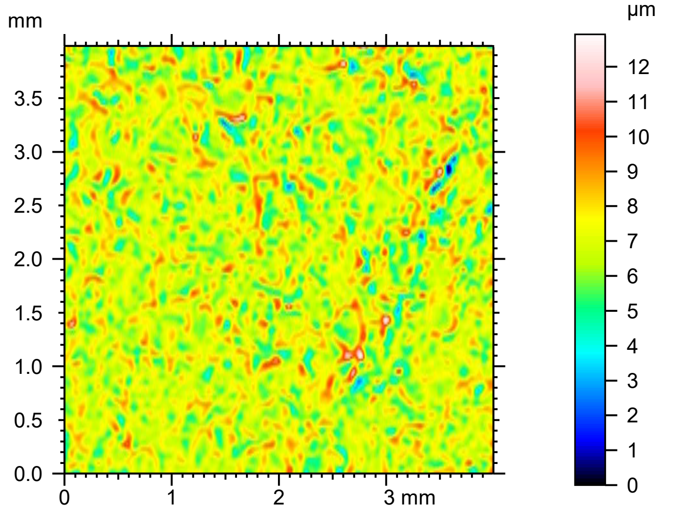

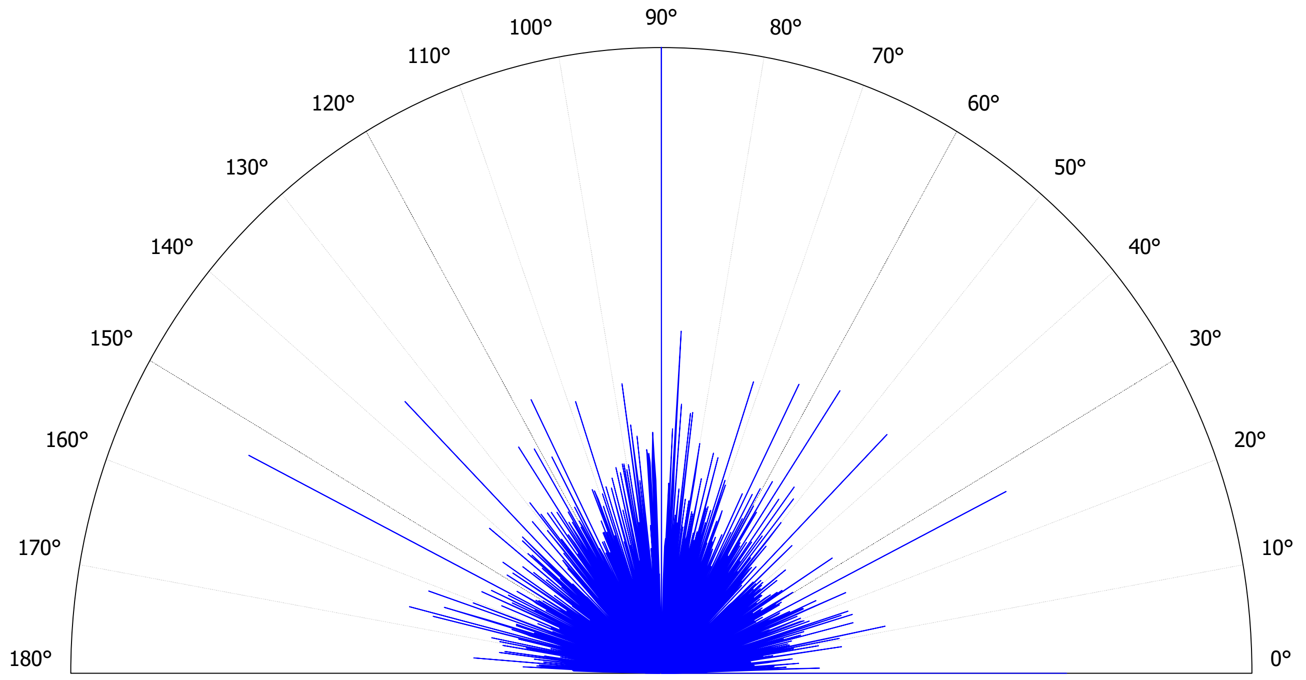

**Parameters**	Value	Unit
Isotropy	72.9	%
First Direction	90.0	°
Second Direction	154	°
Third Direction	0.113	°

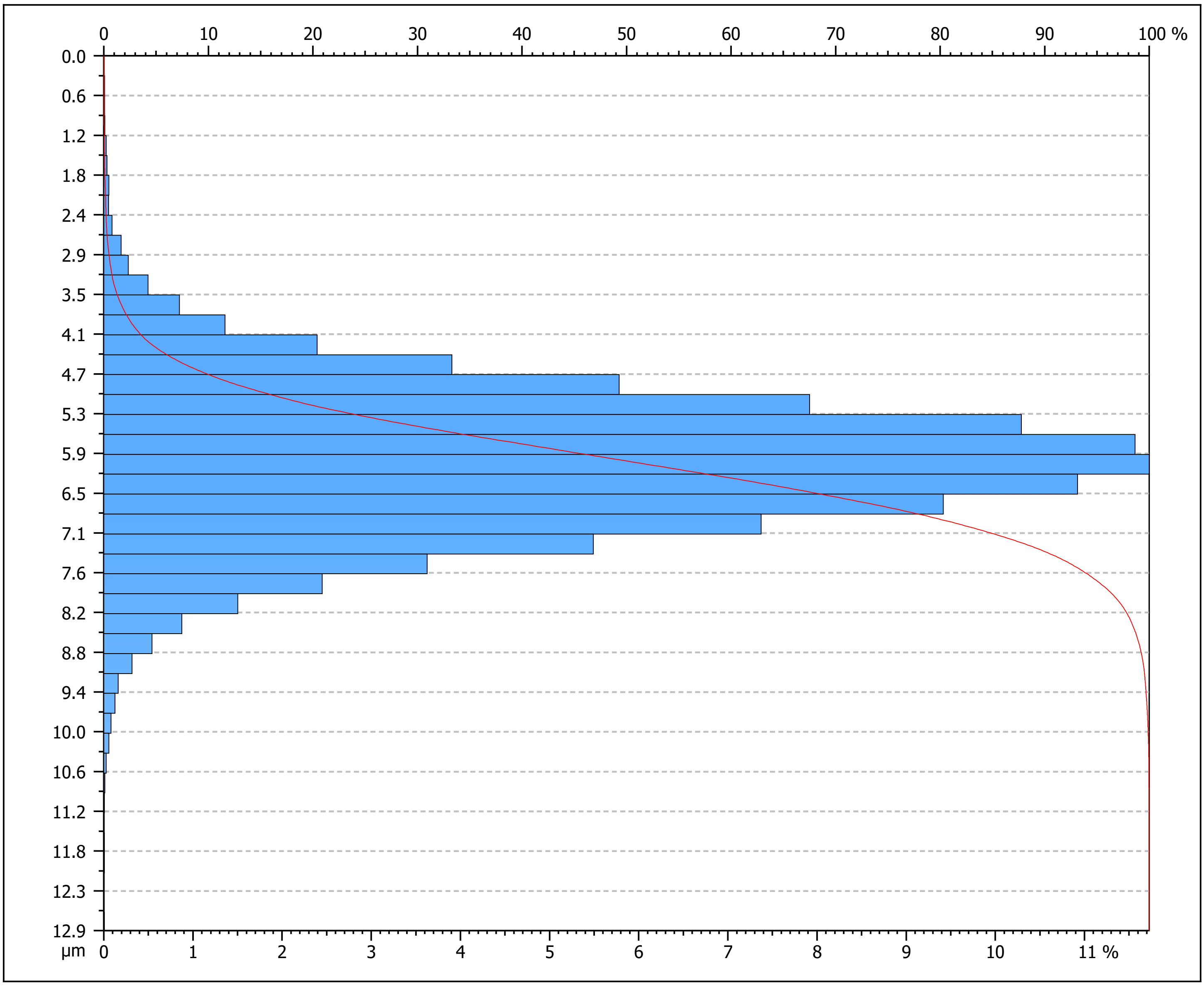
0
10
20
30
40
50
60
70
80
90
100 %
0.0
0.6
1.2
1.8
2.4
2.9
3.5
4.1
4.7
5.3
5.9
6.5
7.1
7.6
8.2
8.8
9.4
10.0
10.6
11.2
11.8
12.3
12.9
µm
0
1
2
3
4
5
6
7
8
9
10
11 %
