# Supplementary material for: Discrimination of Surface Topographies Created by Two-Stage Process by Means of Multiscale Analysis
Source: Materials (Basel). 2021 Nov 20;14(22):7044. doi: 10.3390/ma14227044 (PMC8624860; doi:10.3390/ma14227044)
Supplement: Supplementary file 1 [file materials-14-07044-s001.zip › supplementary materials/rose plots and heights distribution/RingA_inner_1 80-180.pdf]

Identity card	
Name:	P1A_wewnetrzna_1 80-180 > P1A_wewnetrzna_1 80-180 > P1A_wewnetrzna_1 80-180 > P1A_wewnetrzna_1 80-180

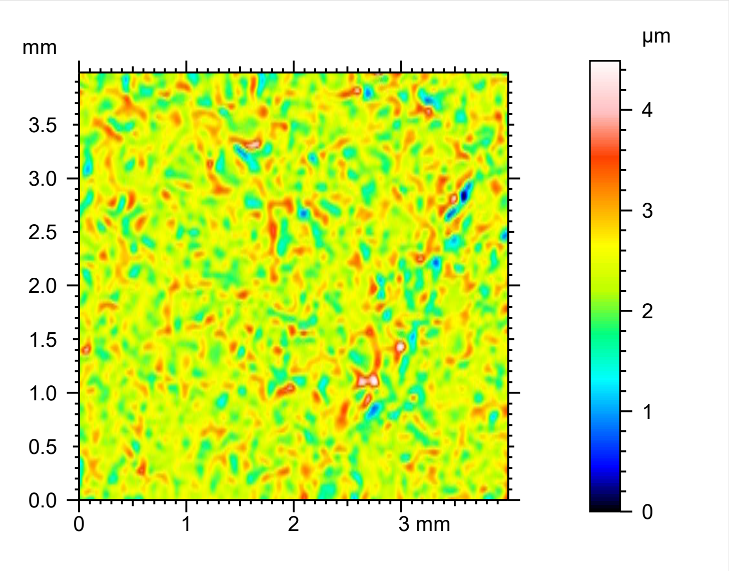

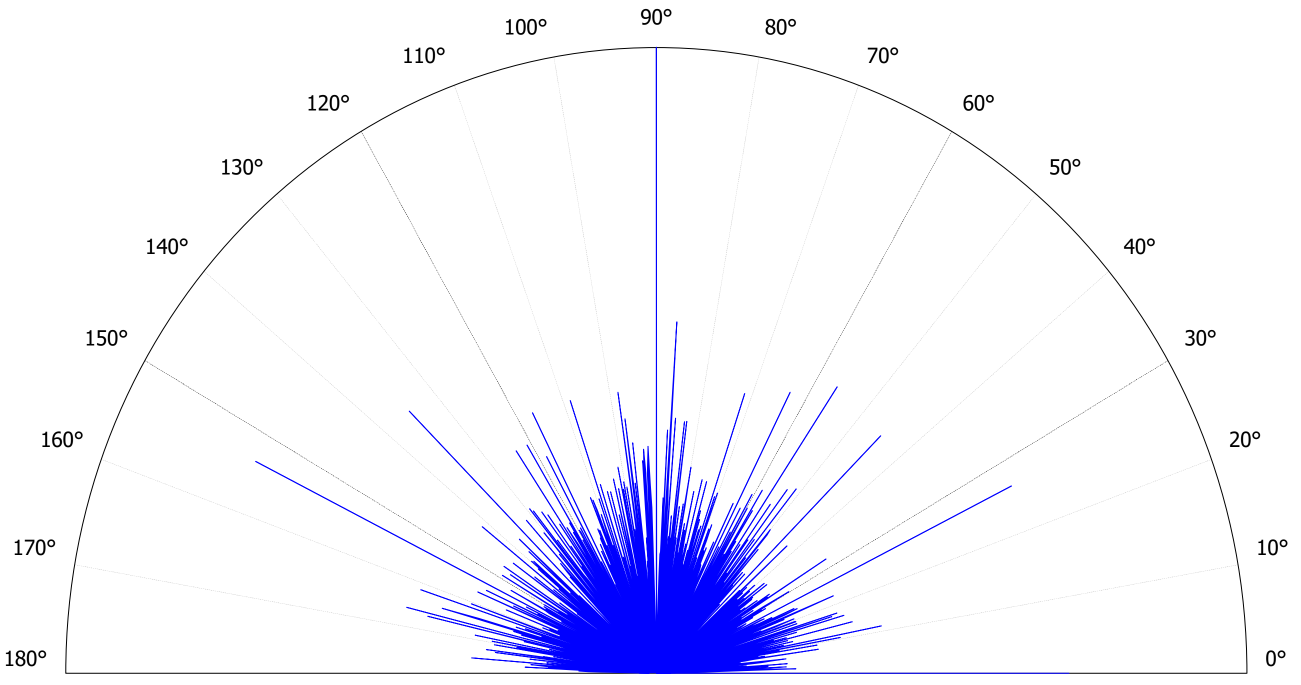

Parameters	Value	Unit
Isotropy	75.9	%
First Direction	90.0	°
Second Direction	154	°
Third Direction	0.108	°

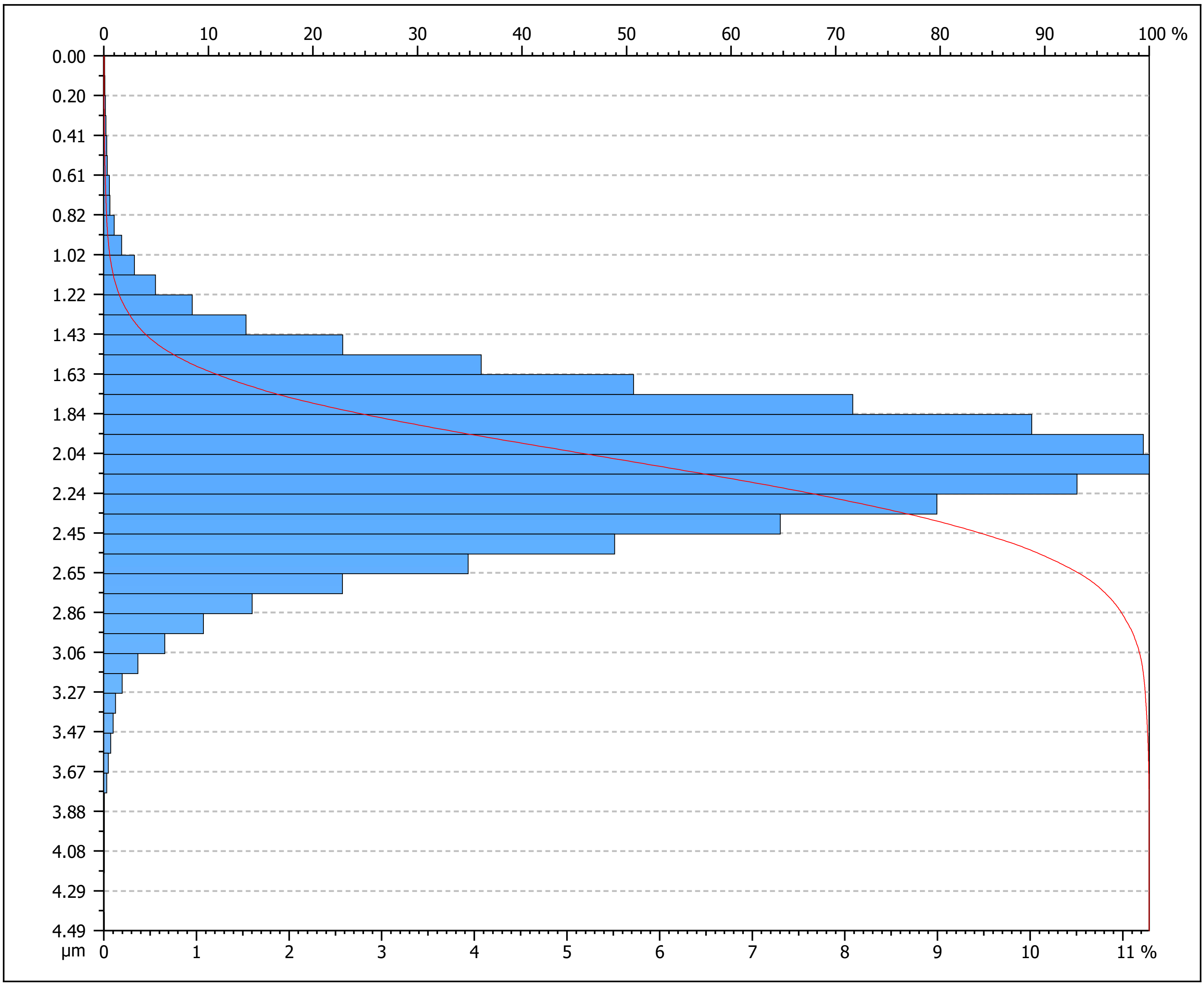

0
10
20
30
40
50
60
70
80
90
100 %
0.00
0.20
0.41
0.61
0.82
1.02
1.22
1.43
1.63
1.84
2.04
2.24
2.45
2.65
2.86
3.06
3.27
3.47
3.67
3.88
4.08
4.29
4.49
µm
0
1
2
3
4
5
6
7
8
9
10
11 %
