# Supplementary material for: Discrimination of Surface Topographies Created by Two-Stage Process by Means of Multiscale Analysis
Source: Materials (Basel). 2021 Nov 20;14(22):7044. doi: 10.3390/ma14227044 (PMC8624860; doi:10.3390/ma14227044)
Supplement: Supplementary file 1 [file materials-14-07044-s001.zip › supplementary materials/rose plots and heights distribution/RingA_lower_1 80-180.pdf]

Identity card	
Name:	P1A_boczna_2_1 80-180 > P1A_boczna_2_1 80-180 > P1A_boczna_2_1 80-180 > P1A_boczna_2_1 80-180

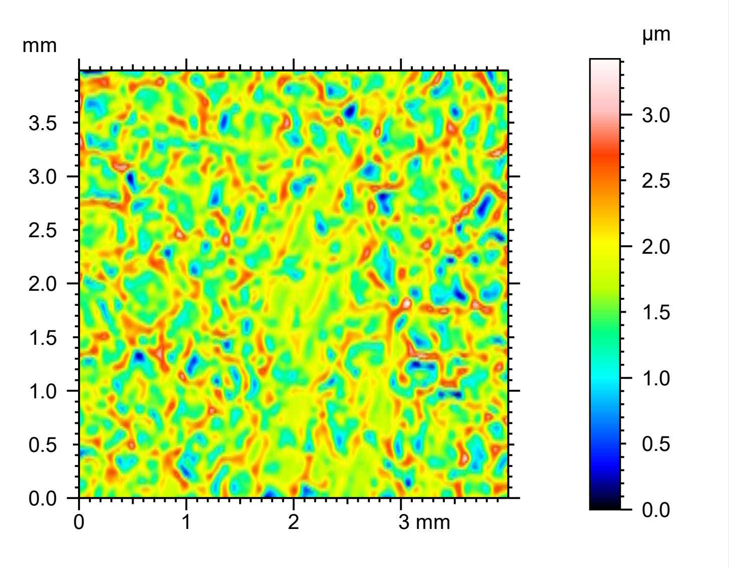

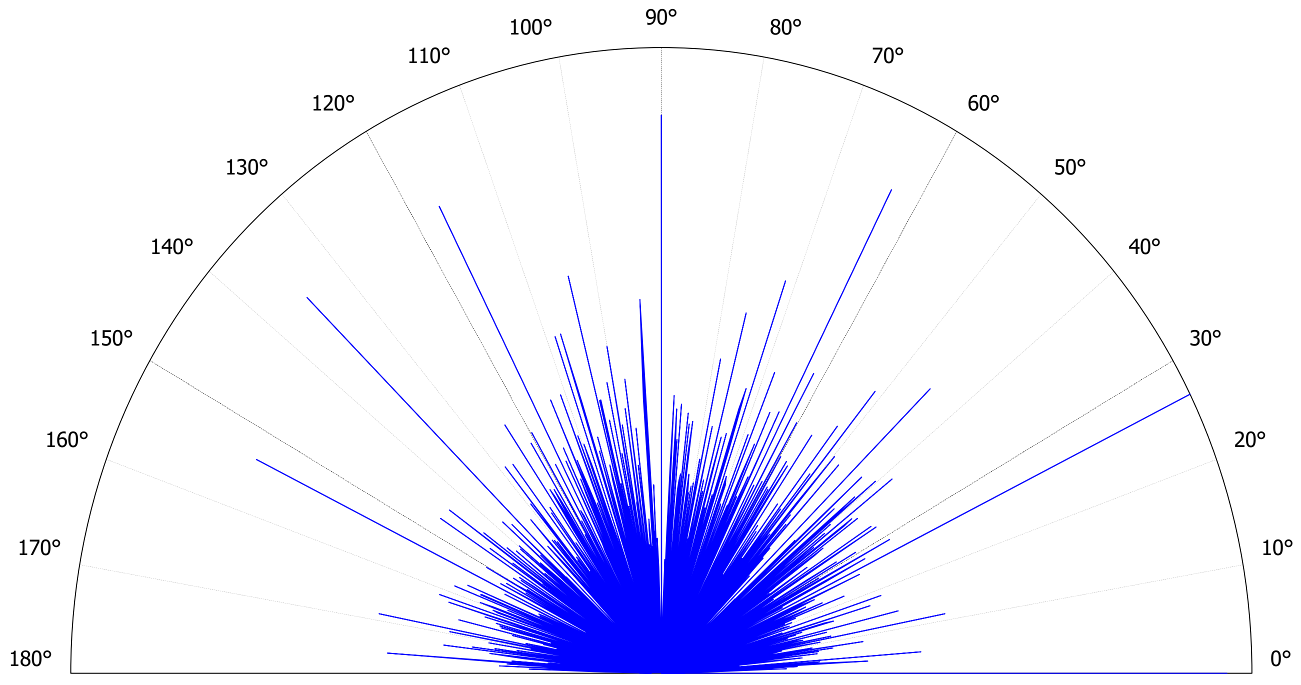

Parameters	Value	Unit
Isotropy	81.0	%
First Direction	26.5	°
Second Direction	0.107	°
Third Direction	90.0	°

MountainsMap® Premium 7.4.8083

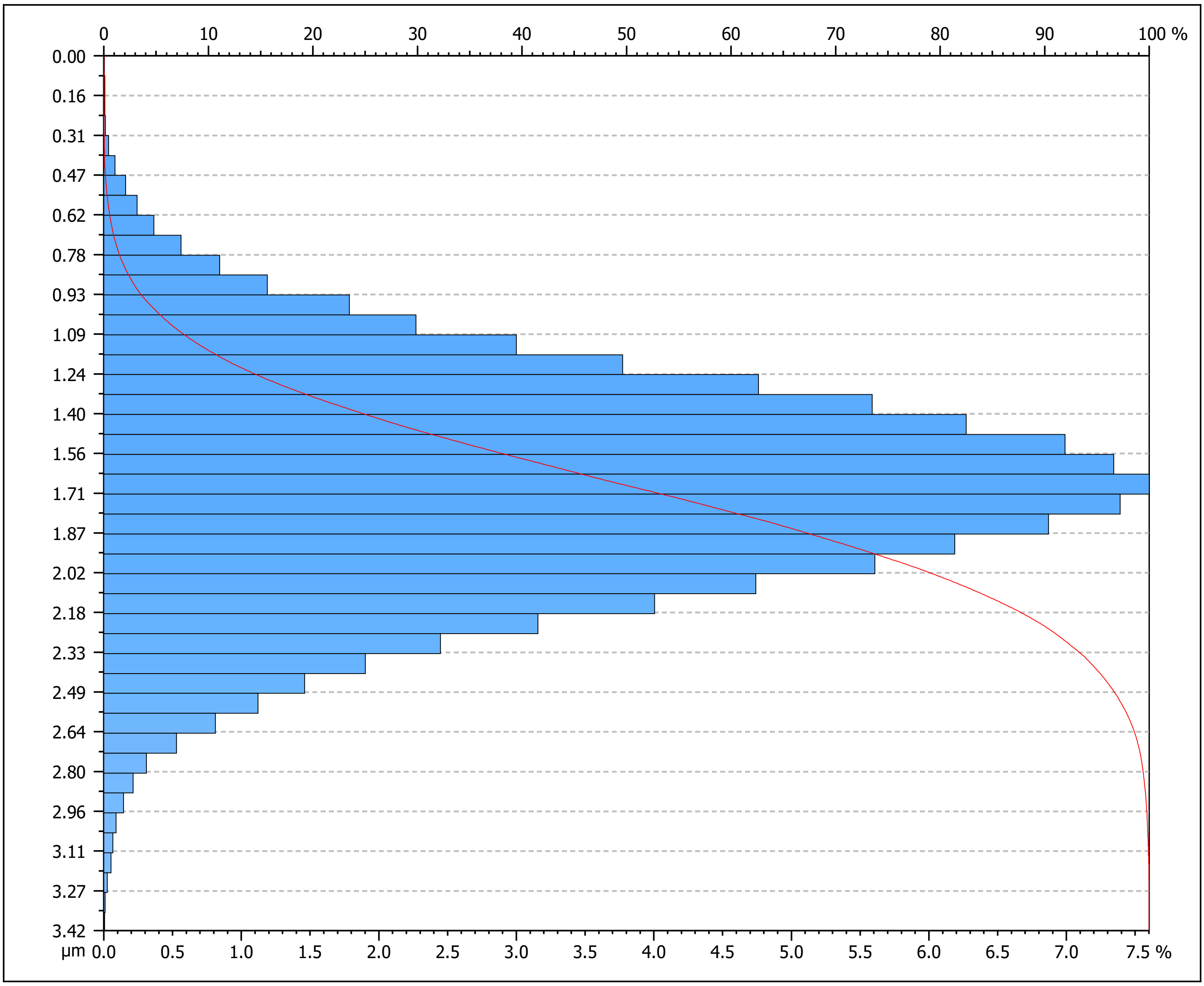
0 10 20 30 40 50 60 70 80 90 100 %
0.00 0.16 0.31 0.47 0.62 0.78 0.93 1.09 1.24 1.40 1.56 1.71 1.87 2.02 2.18 2.33 2.49 2.64 2.80 2.96 3.11 3.27 3.42
µm 0.0 0.5 1.0 1.5 2.0 2.5 3.0 3.5 4.0 4.5 5.0 5.5 6.0 6.5 7.0 7.5 %
