# Supplementary material for: Discrimination of Surface Topographies Created by Two-Stage Process by Means of Multiscale Analysis
Source: Materials (Basel). 2021 Nov 20;14(22):7044. doi: 10.3390/ma14227044 (PMC8624860; doi:10.3390/ma14227044)
Supplement: Supplementary file 1 [file materials-14-07044-s001.zip › supplementary materials/rose plots and heights distribution/RingA_upper_1 145-195.pdf]

Identity card	
Name:	P1A_boczna_1_1 145-195 > P1A_boczna_1_1 145-195 > P1A_boczna_1_1 145-195 > P1A_boczna_1_1 145-195

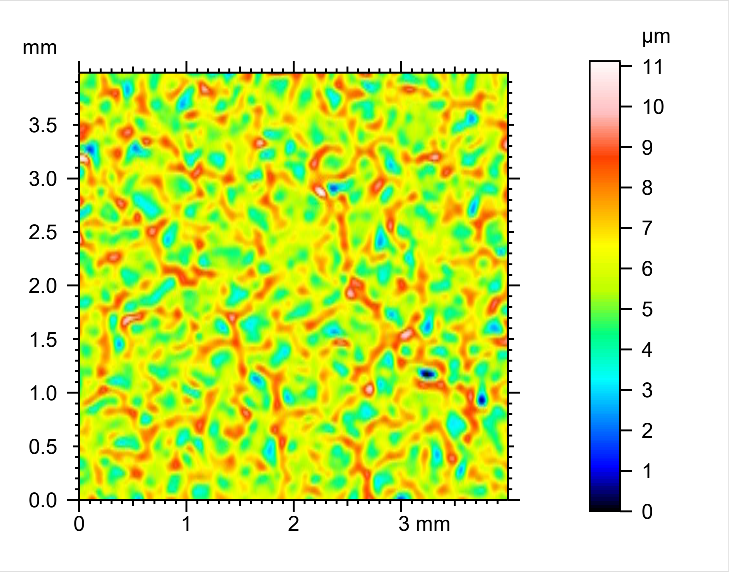

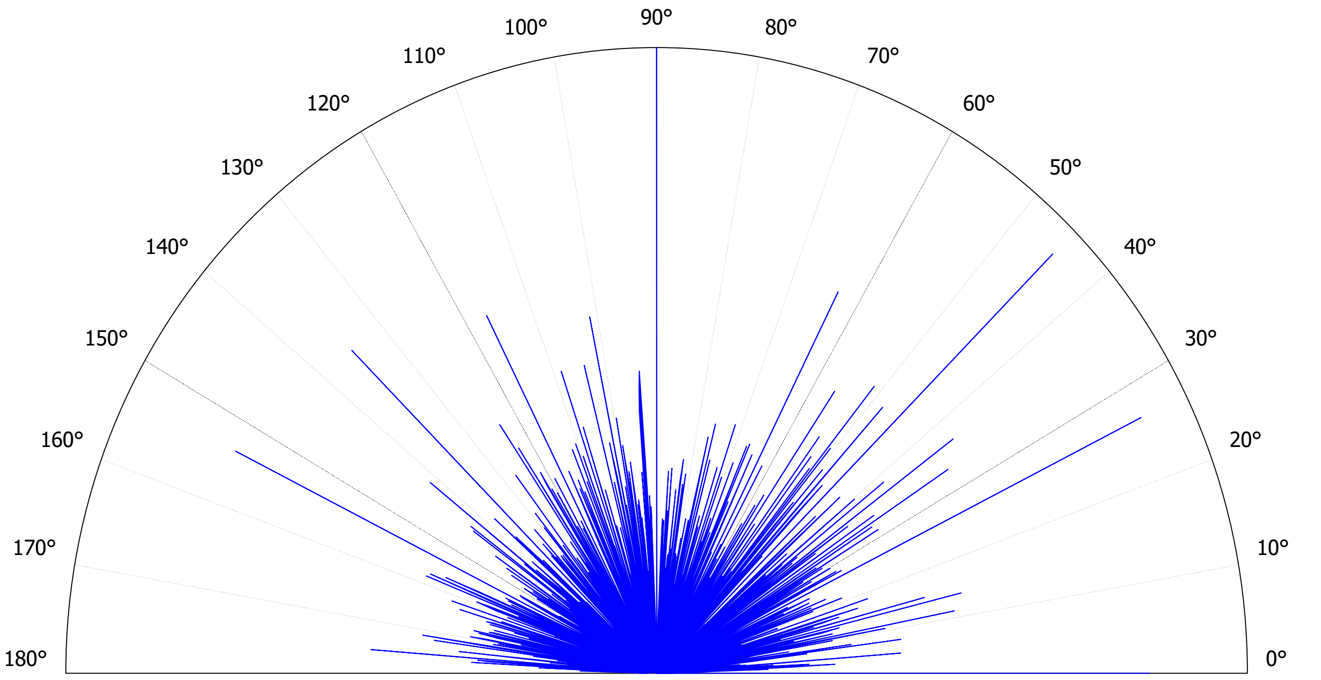

Parameters	Value	Unit
Isotropy	84.3	%
First Direction	90.0	°
Second Direction	45.0	°
Third Direction	26.5	°

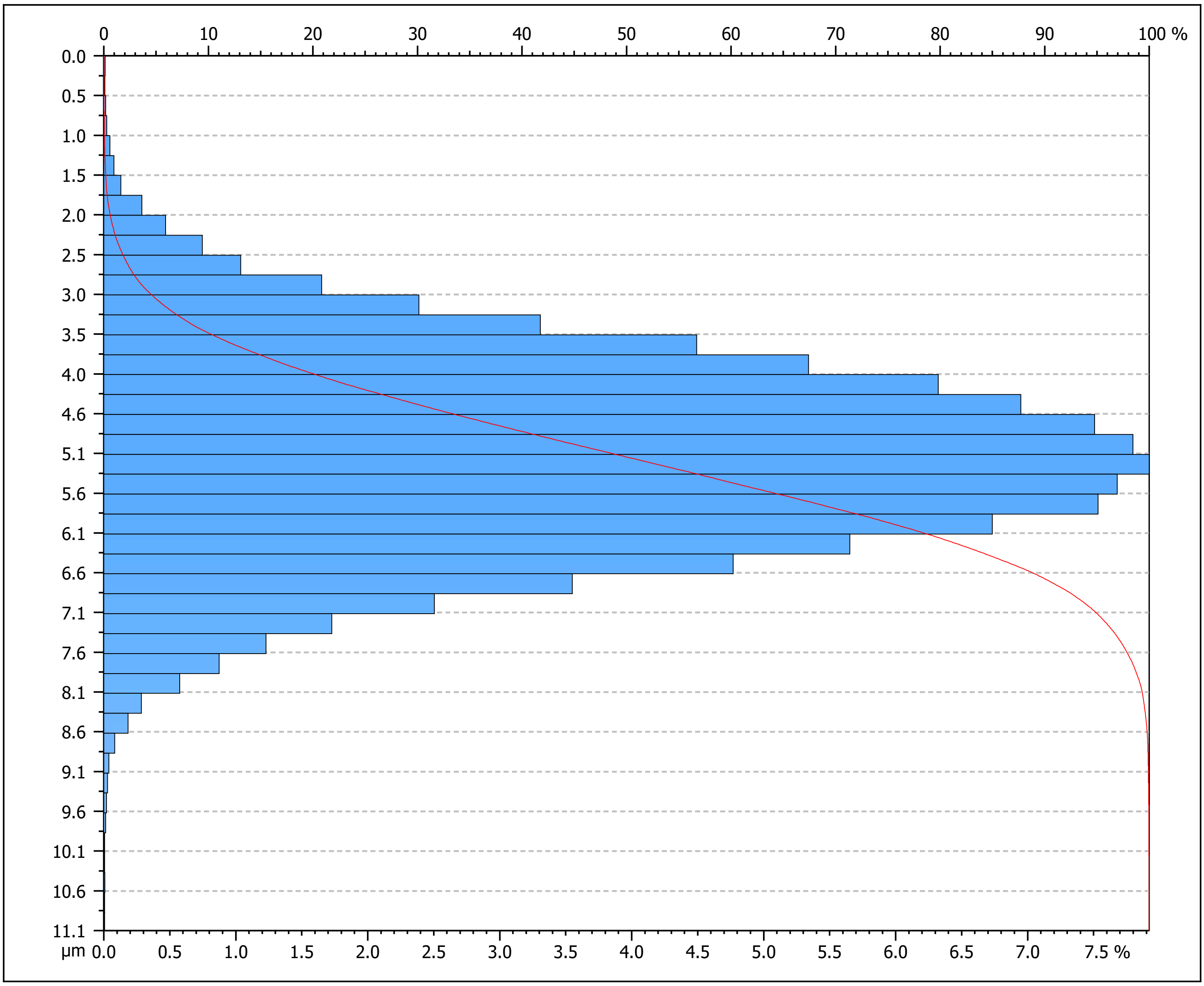
0 10 20 30 40 50 60 70 80 90 100 %
0.0
0.5
1.0
1.5
2.0
2.5
3.0
3.5
4.0
4.6
5.1
5.6
6.1
6.6
7.1
7.6
8.1
8.6
9.1
9.6
10.1
10.6
11.1
µm
0.0 0.5 1.0 1.5 2.0 2.5 3.0 3.5 4.0 4.5 5.0 5.5 6.0 6.5 7.0 7.5 %
