# Supplementary material for: Discrimination of Surface Topographies Created by Two-Stage Process by Means of Multiscale Analysis
Source: Materials (Basel). 2021 Nov 20;14(22):7044. doi: 10.3390/ma14227044 (PMC8624860; doi:10.3390/ma14227044)
Supplement: Supplementary file 1 [file materials-14-07044-s001.zip › supplementary materials/rose plots and heights distribution/RingA_upper_1 80-180.pdf]

Identity card

Name:	P1A_boczna_1_1 80-180 > P1A_boczna_1_1 80-180 > P1A_boczna_1_1 80-180 > P1A_boczna_1_1 80-180

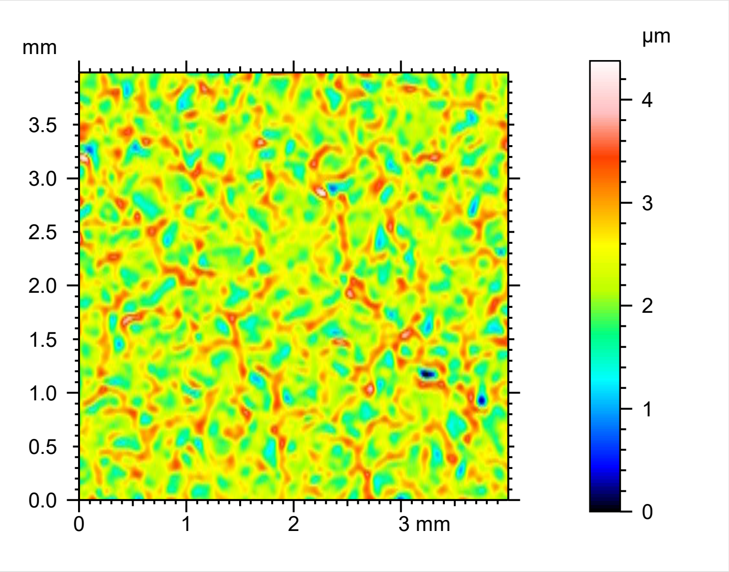

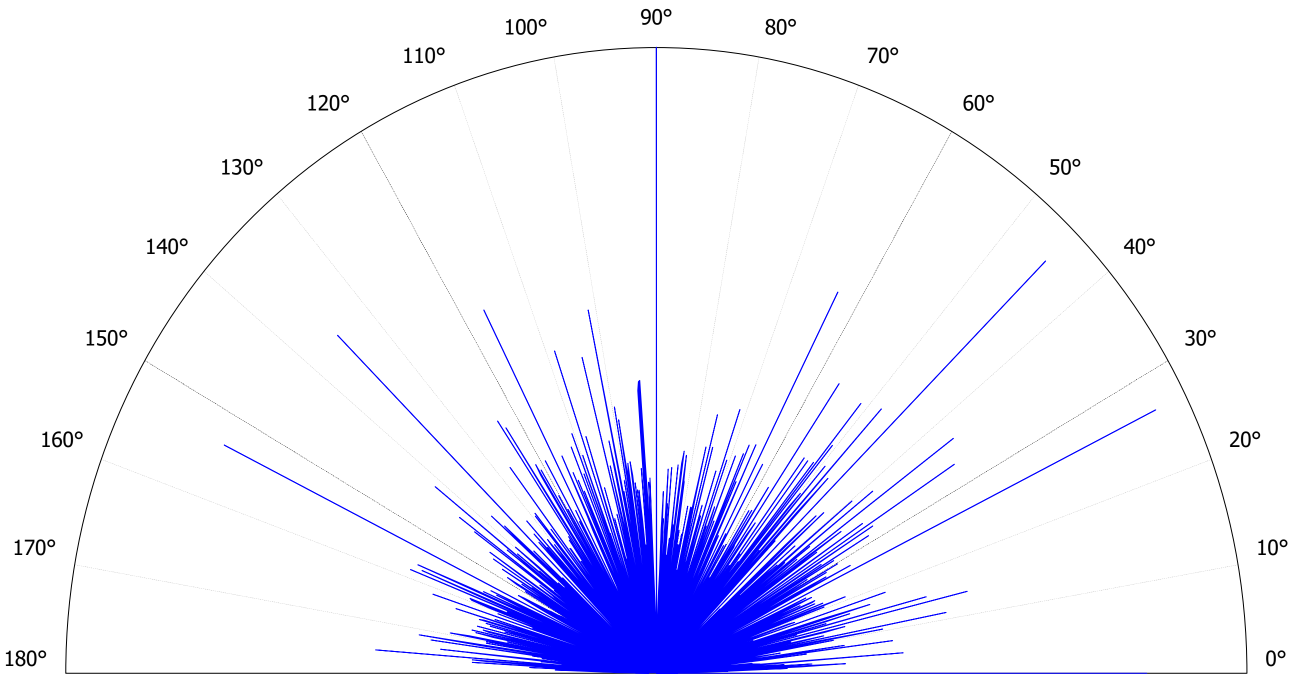

Parameters	Value	Unit
Isotropy	83.8	%
First Direction	90.0	°
Second Direction	26.5	°
Third Direction	45.0	°

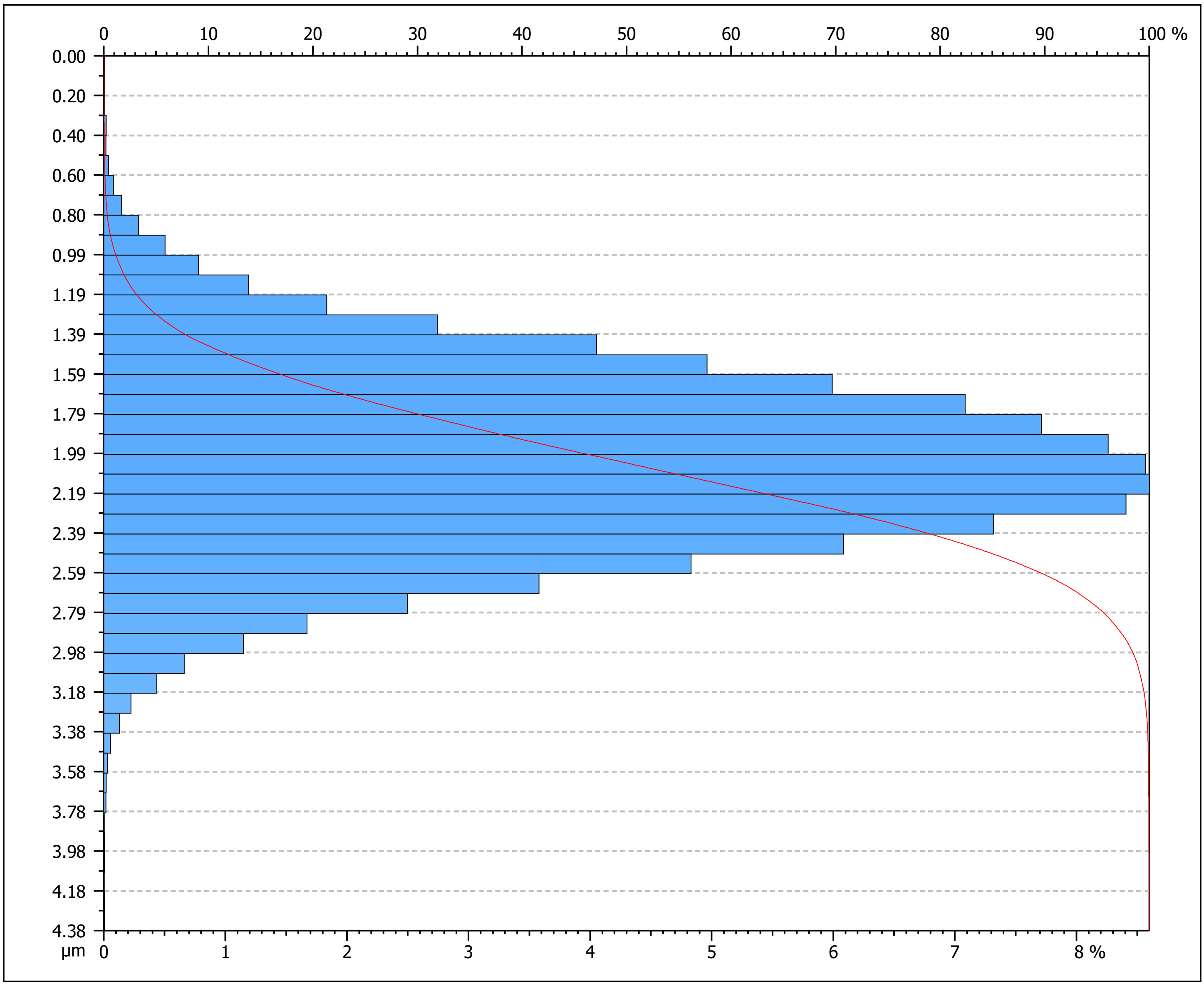

0
10
20
30
40
50
60
70
80
90
100 %
0.00
0.20
0.40
0.60
0.80
0.99
1.19
1.39
1.59
1.79
1.99
2.19
2.39
2.59
2.79
2.98
3.18
3.38
3.58
3.78
3.98
4.18
4.38
µm
0
1
2
3
4
5
6
7
8 %
