# Supplementary material for: Discrimination of Surface Topographies Created by Two-Stage Process by Means of Multiscale Analysis
Source: Materials (Basel). 2021 Nov 20;14(22):7044. doi: 10.3390/ma14227044 (PMC8624860; doi:10.3390/ma14227044)
Supplement: Supplementary file 1 [file materials-14-07044-s001.zip › supplementary materials/rose plots and heights distribution/RingB_inner_1 145-195.pdf]

Identity card

Name:	P4C_wewnetrzna_1 145-195 > P4C_wewnetrzna_1 145-195 > P4C_wewnetrzna_1 145-195 > P4C_wewnetrzna_1 145-195

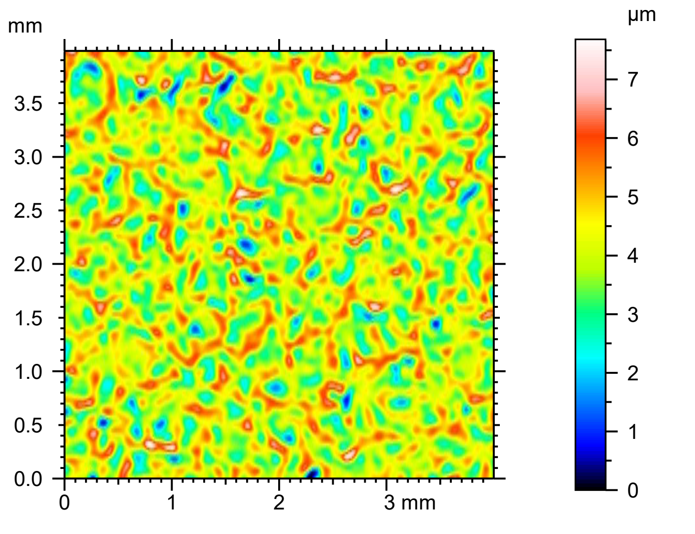

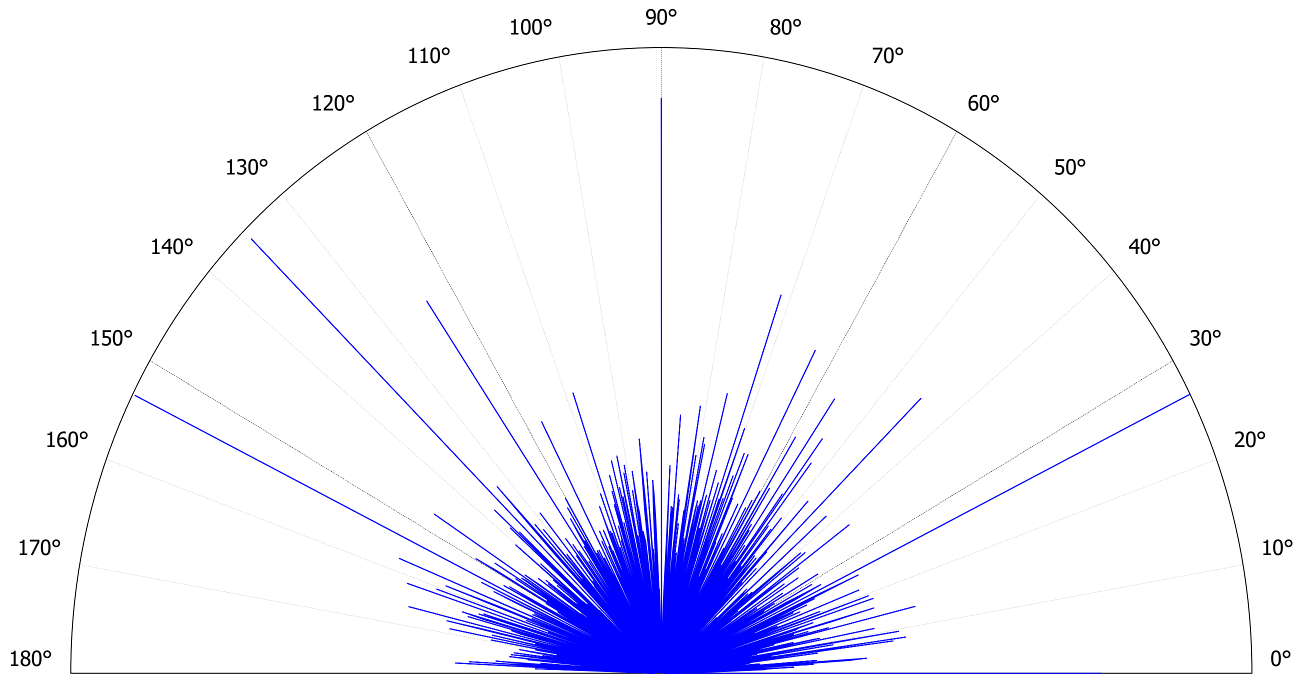

Parameters	Value	Unit
Isotropy	82.5	%
First Direction	26.5	°
Second Direction	153	°
Third Direction	135	°

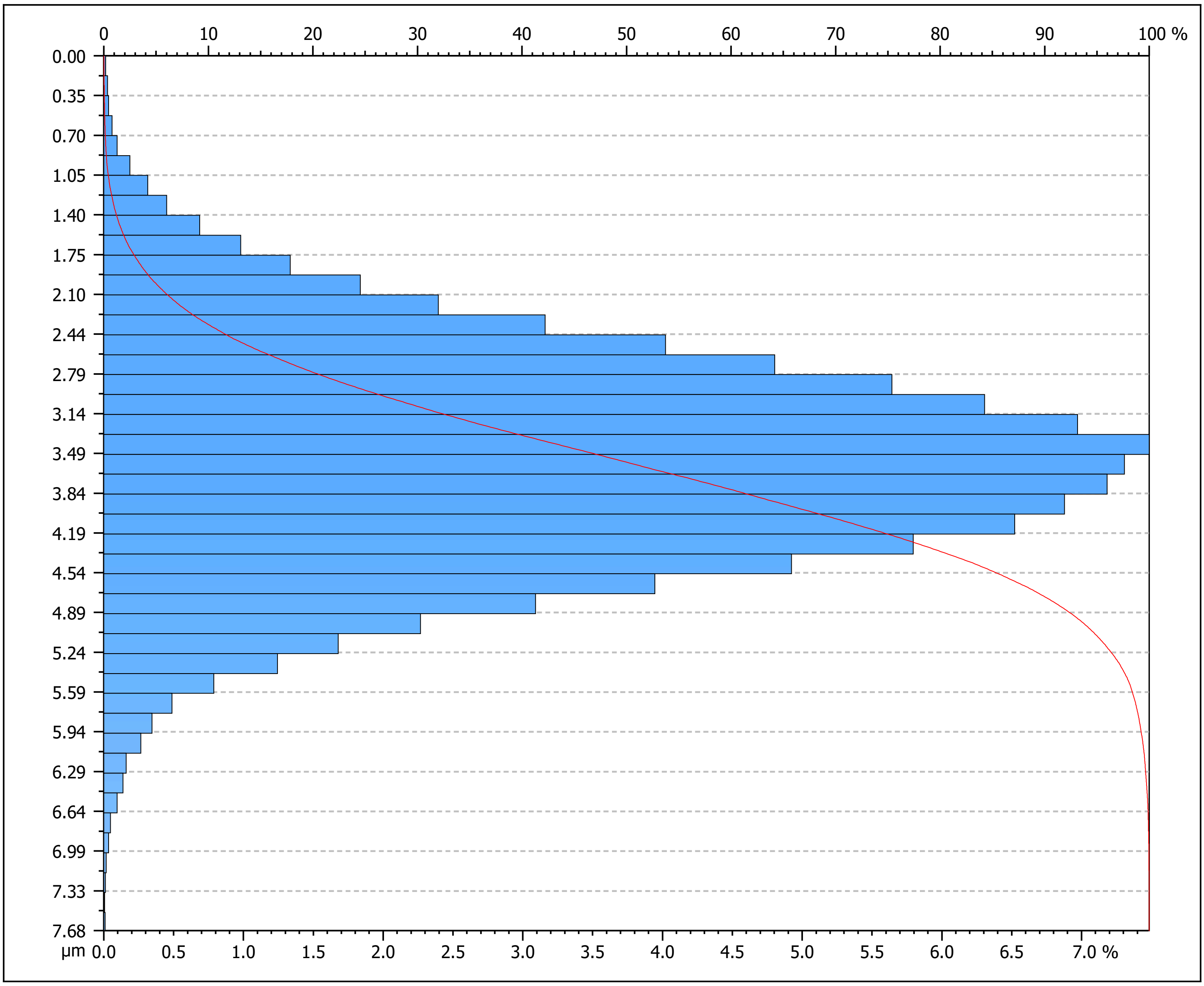
0
10
20
30
40
50
60
70
80
90
100 %
0.00
0.35
0.70
1.05
1.40
1.75
2.10
2.44
2.79
3.14
3.49
3.84
4.19
4.54
4.89
5.24
5.59
5.94
6.29
6.64
6.99
7.33
7.68
µm
0.0
0.5
1.0
1.5
2.0
2.5
3.0
3.5
4.0
4.5
5.0
5.5
6.0
6.5
7.0 %
