# Supplementary material for: Discrimination of Surface Topographies Created by Two-Stage Process by Means of Multiscale Analysis
Source: Materials (Basel). 2021 Nov 20;14(22):7044. doi: 10.3390/ma14227044 (PMC8624860; doi:10.3390/ma14227044)
Supplement: Supplementary file 1 [file materials-14-07044-s001.zip › supplementary materials/rose plots and heights distribution/RingB_inner_1 180-198.pdf]

Identity card

Name:	P4C_wewnetrzna_1 180-198 > P4C_wewnetrzna_1 180-198 > P4C_wewnetrzna_1 180-198 > P4C_wewnetrzna_1 180-198

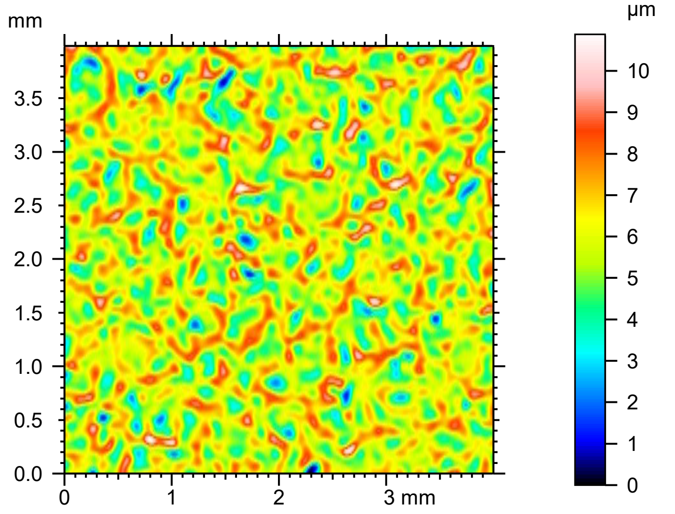

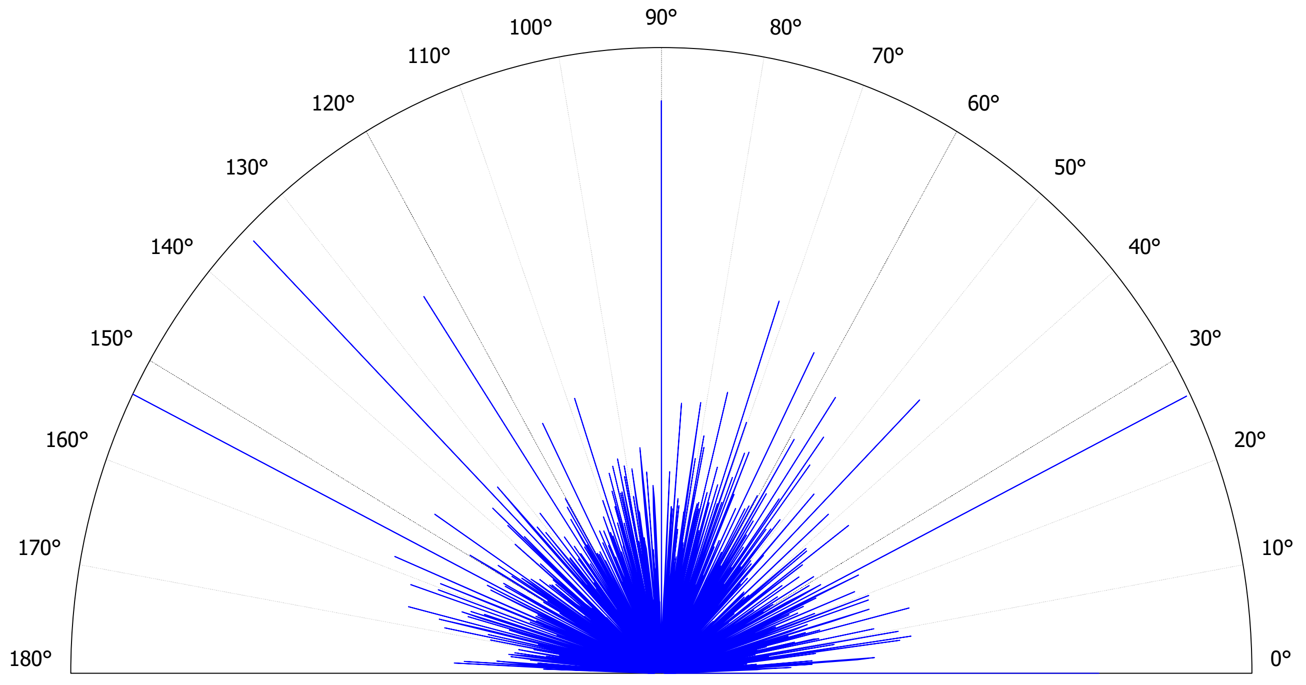

Parameters	Value	Unit
Isotropy	81.8	%
First Direction	153	°
Second Direction	26.5	°
Third Direction	135	°

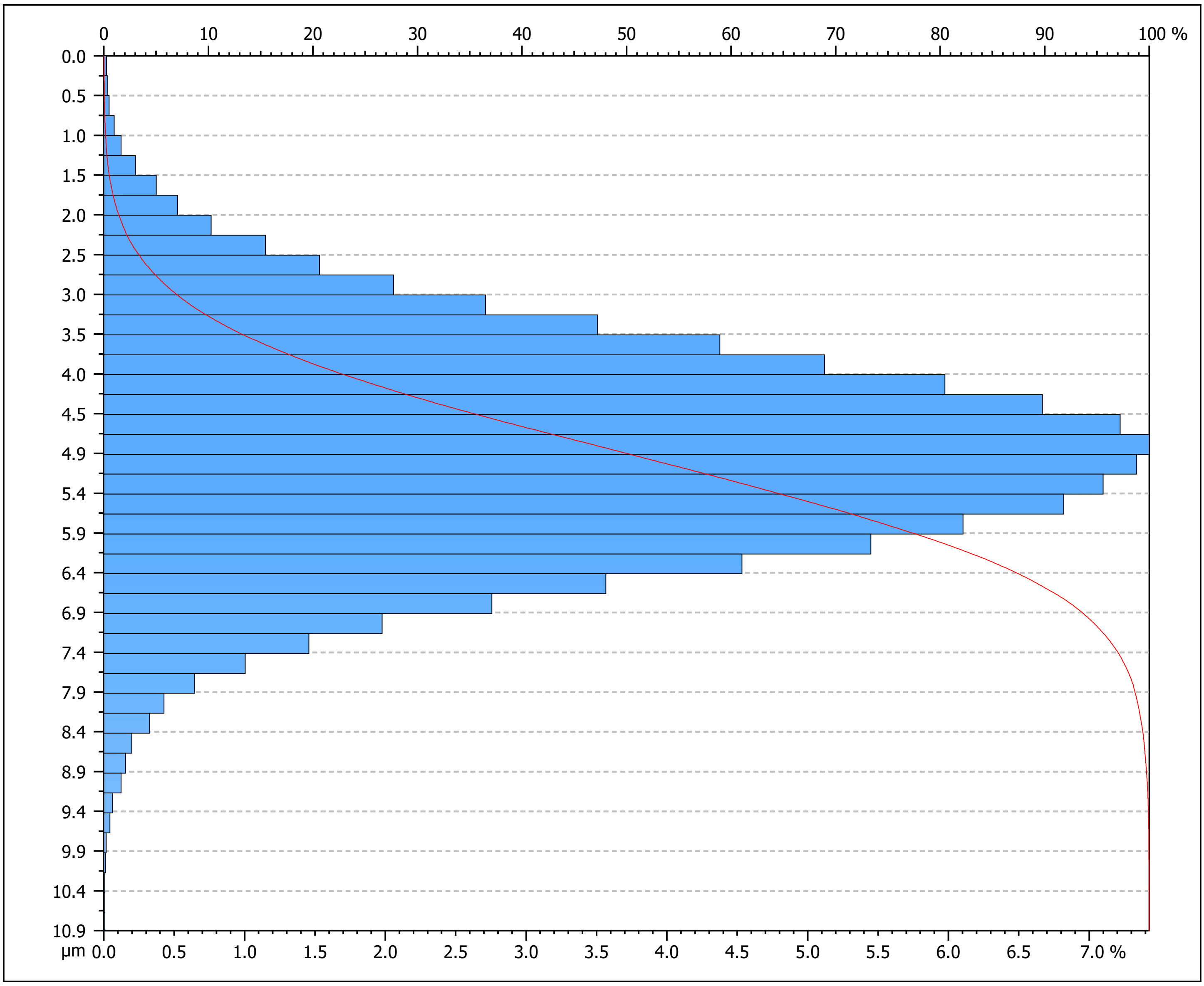

0 10 20 30 40 50 60 70 80 90 100 %
0.0 0.5 1.0 1.5 2.0 2.5 3.0 3.5 4.0 4.5 4.9 5.4 5.9 6.4 6.9 7.4 7.9 8.4 8.9 9.4 9.9 10.4 10.9
µm 0.0 0.5 1.0 1.5 2.0 2.5 3.0 3.5 4.0 4.5 5.0 5.5 6.0 6.5 7.0 %
