# Supplementary material for: Discrimination of Surface Topographies Created by Two-Stage Process by Means of Multiscale Analysis
Source: Materials (Basel). 2021 Nov 20;14(22):7044. doi: 10.3390/ma14227044 (PMC8624860; doi:10.3390/ma14227044)
Supplement: Supplementary file 1 [file materials-14-07044-s001.zip › supplementary materials/rose plots and heights distribution/RingB_lower_1 180-198.pdf]

Identity card

Name:	P4C_boczna_2_1 180-198 > P4C_boczna_2_1 180-198 > P4C_boczna_2_1 180-198 > P4C_boczna_2_1 180-198

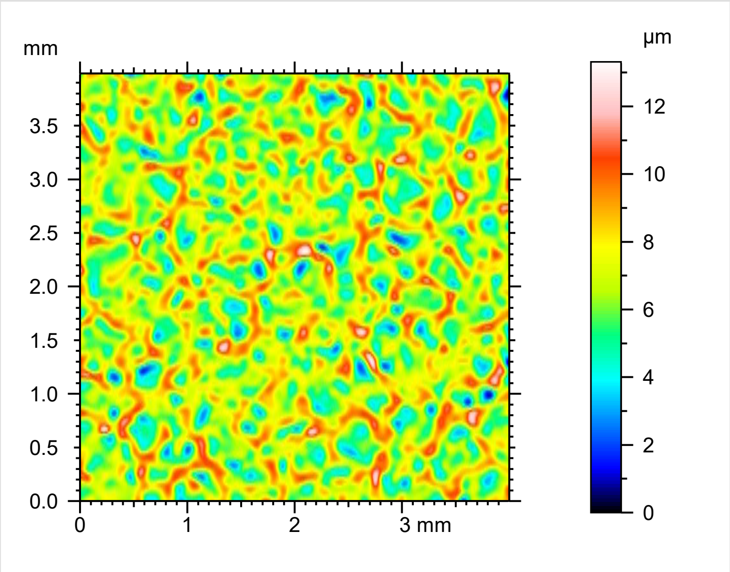

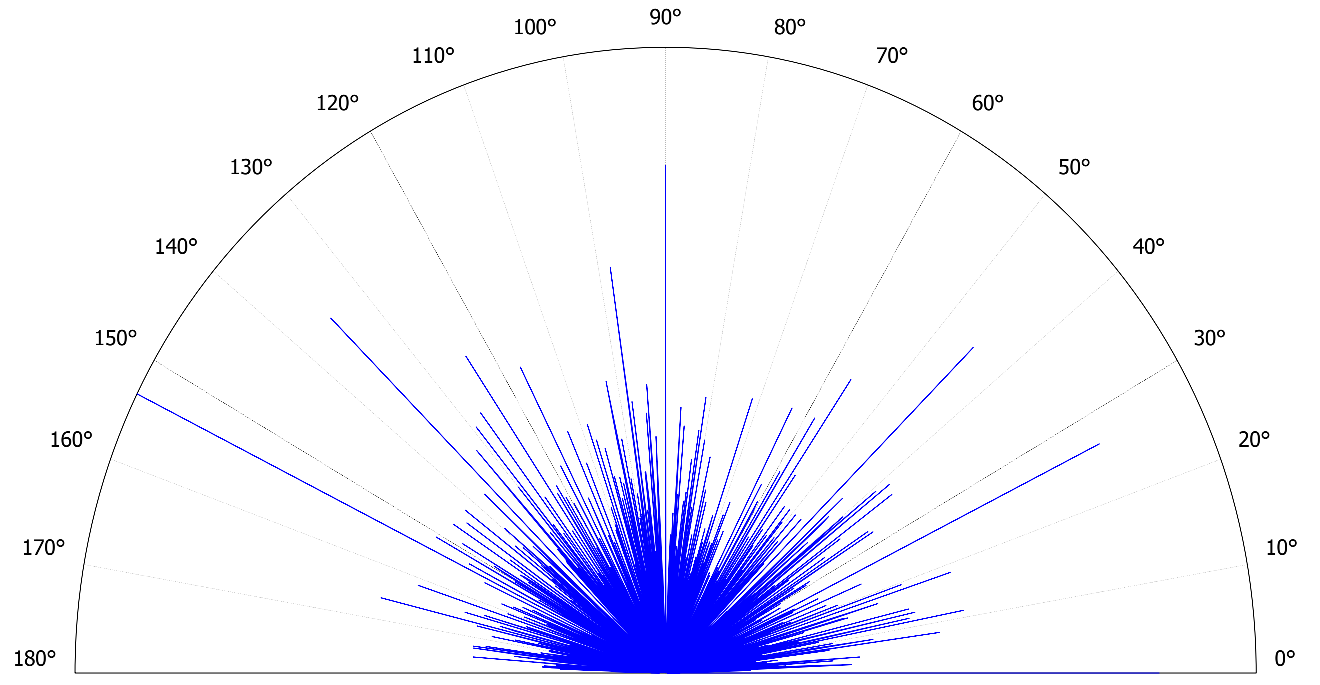

Parameters	Value	Unit
Isotropy	81.6	%
First Direction	154	°
Second Direction	0.110	°
Third Direction	26.5	°

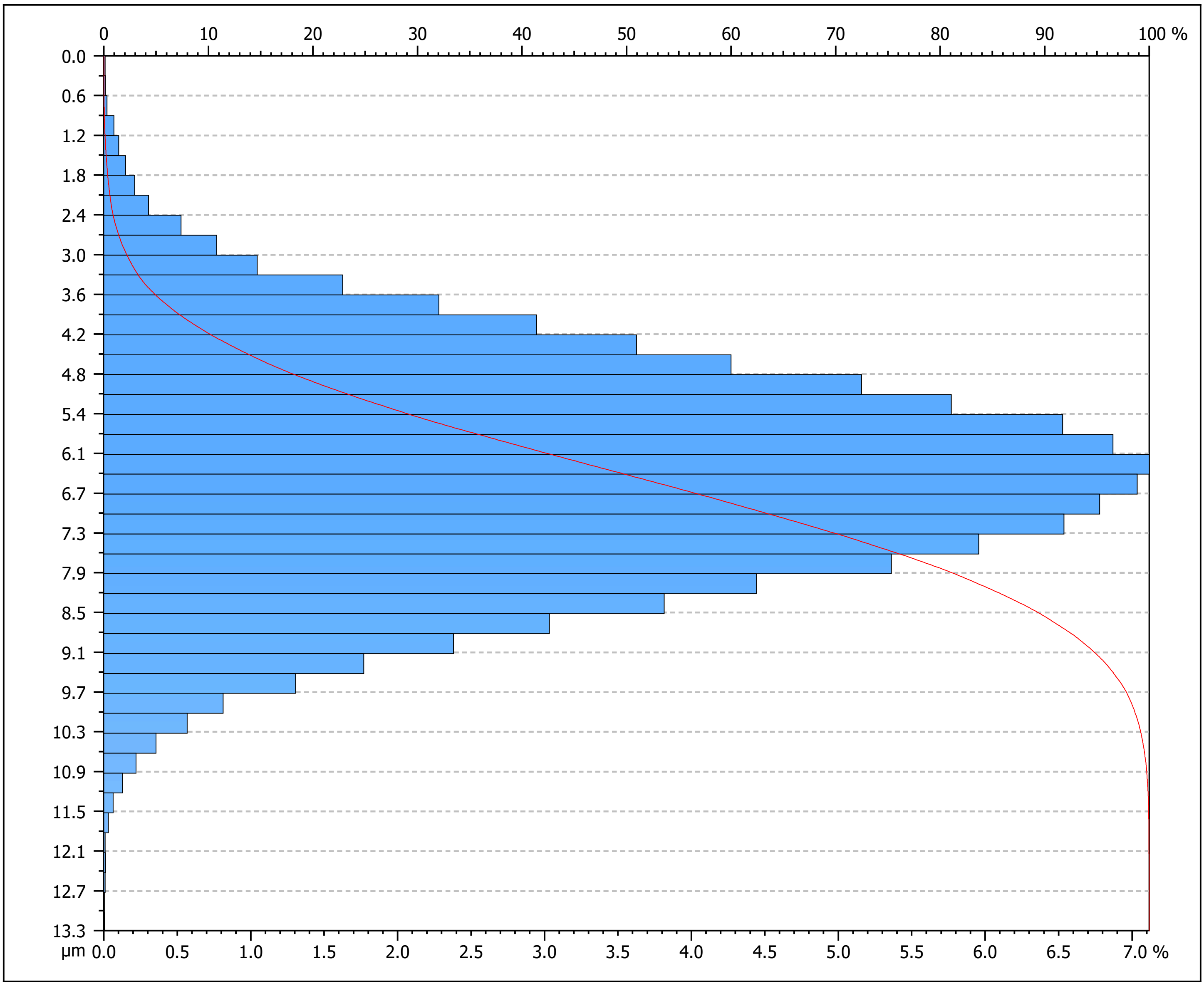
0
10
20
30
40
50
60
70
80
90
100 %
0.0
0.6
1.2
1.8
2.4
3.0
3.6
4.2
4.8
5.4
6.1
6.7
7.3
7.9
8.5
9.1
9.7
10.3
10.9
11.5
12.1
12.7
13.3
µm
0.0
0.5
1.0
1.5
2.0
2.5
3.0
3.5
4.0
4.5
5.0
5.5
6.0
6.5
7.0 %
