# Supplementary material for: Discrimination of Surface Topographies Created by Two-Stage Process by Means of Multiscale Analysis
Source: Materials (Basel). 2021 Nov 20;14(22):7044. doi: 10.3390/ma14227044 (PMC8624860; doi:10.3390/ma14227044)
Supplement: Supplementary file 1 [file materials-14-07044-s001.zip › supplementary materials/rose plots and heights distribution/RingB_outer_1 105-155.pdf]

Identity card	
Name:	P4C_zewnetrzna_1 105-155 > P4C_zewnetrzna_1 105-155 > P4C_zewnetrzna_1 105-155 > P4C_zewnetrzna_1 105-155

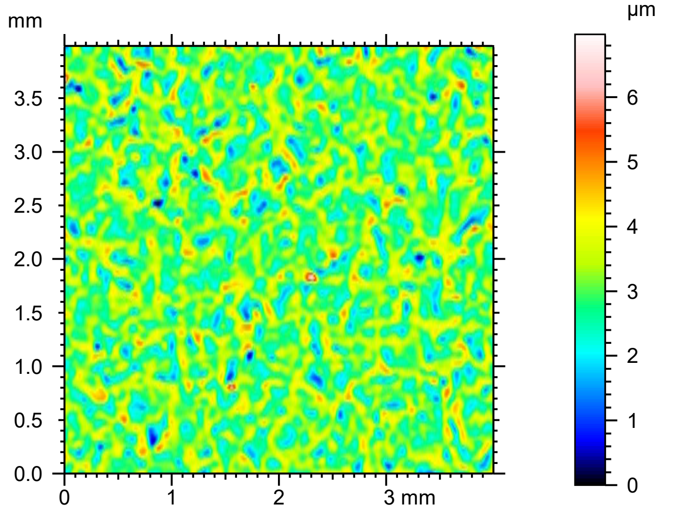

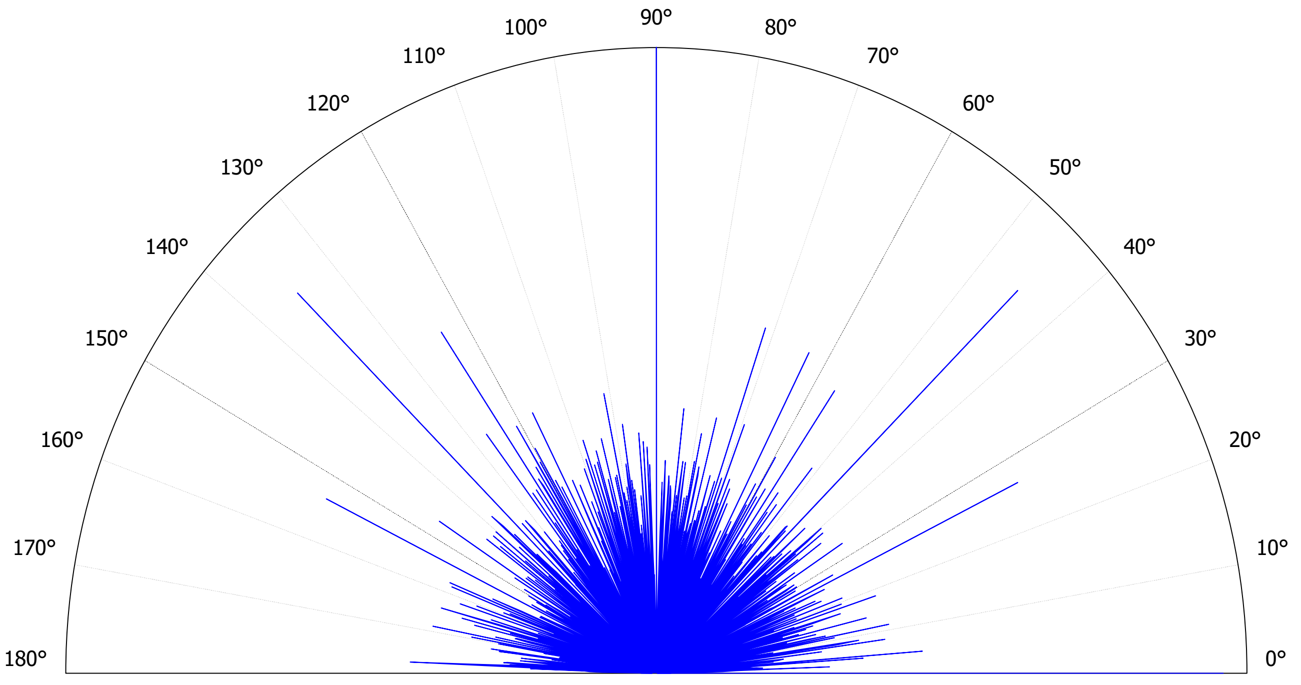

Parameters	Value	Unit
Isotropy	80.2	%
First Direction	90.0	°
Second Direction	0.0933	°
Third Direction	45.0	°

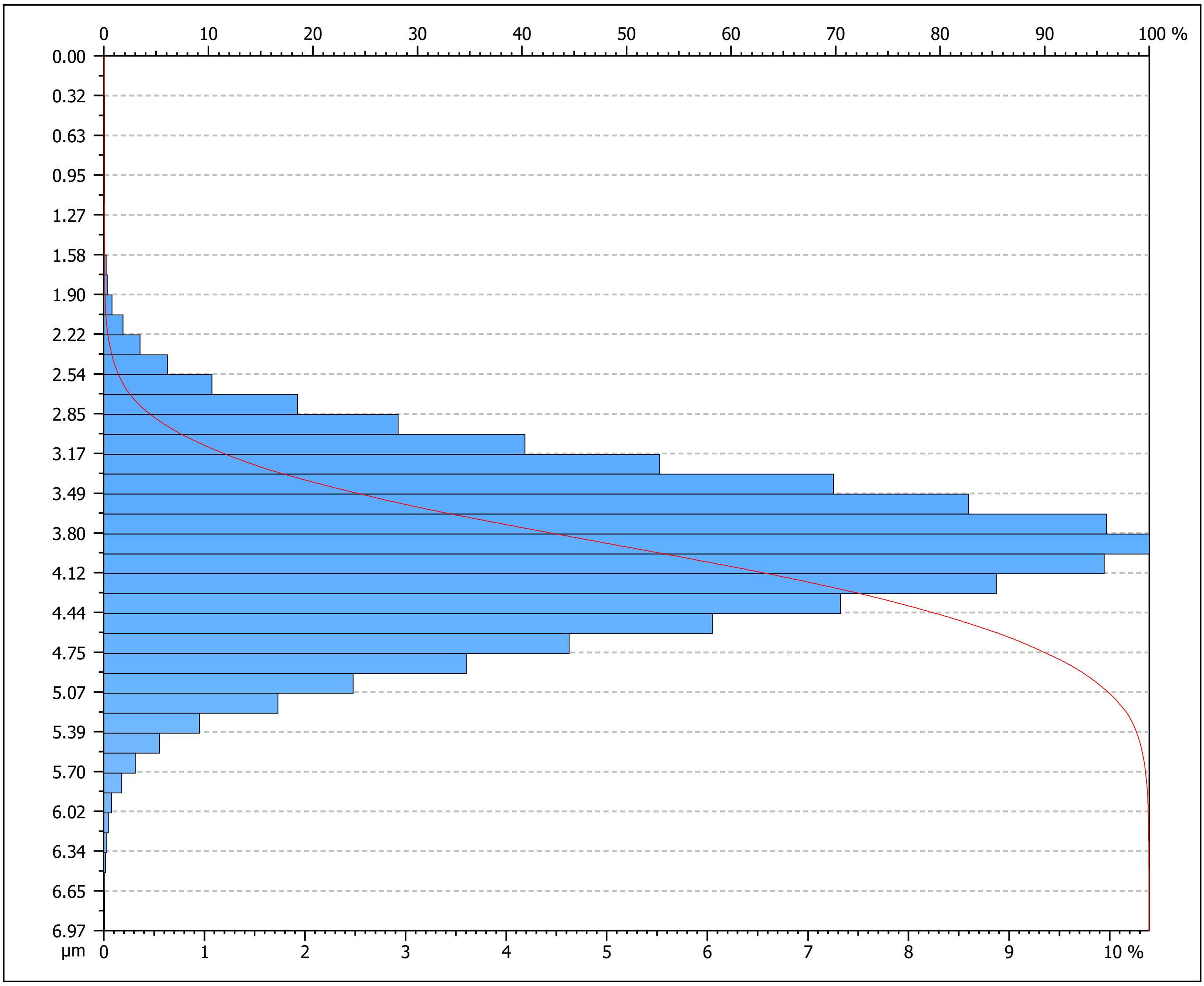
0
10
20
30
40
50
60
70
80
90
100 %
0.00
0.32
0.63
0.95
1.27
1.58
1.90
2.22
2.54
2.85
3.17
3.49
3.80
4.12
4.44
4.75
5.07
5.39
5.70
6.02
6.34
6.65
6.97
µm
0
1
2
3
4
5
6
7
8
9
10 %
