# Supplementary material for: Discrimination of Surface Topographies Created by Two-Stage Process by Means of Multiscale Analysis
Source: Materials (Basel). 2021 Nov 20;14(22):7044. doi: 10.3390/ma14227044 (PMC8624860; doi:10.3390/ma14227044)
Supplement: Supplementary file 1 [file materials-14-07044-s001.zip › supplementary materials/rose plots and heights distribution/RingB_outer_1 65-115.pdf]

Identity card

Name:	P4C_zewnetrzna_1 65-115 > P4C_zewnetrzna_1 65-115 > P4C_zewnetrzna_1 65-115 > P4C_zewnetrzna_1 65-115

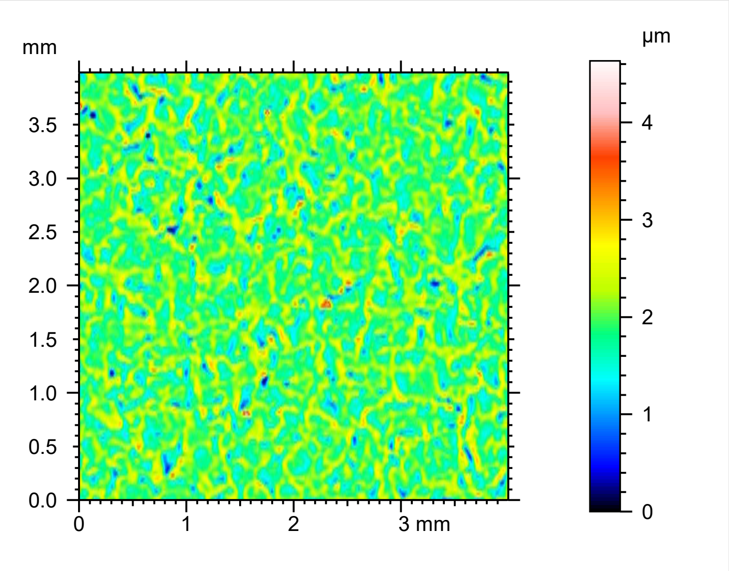

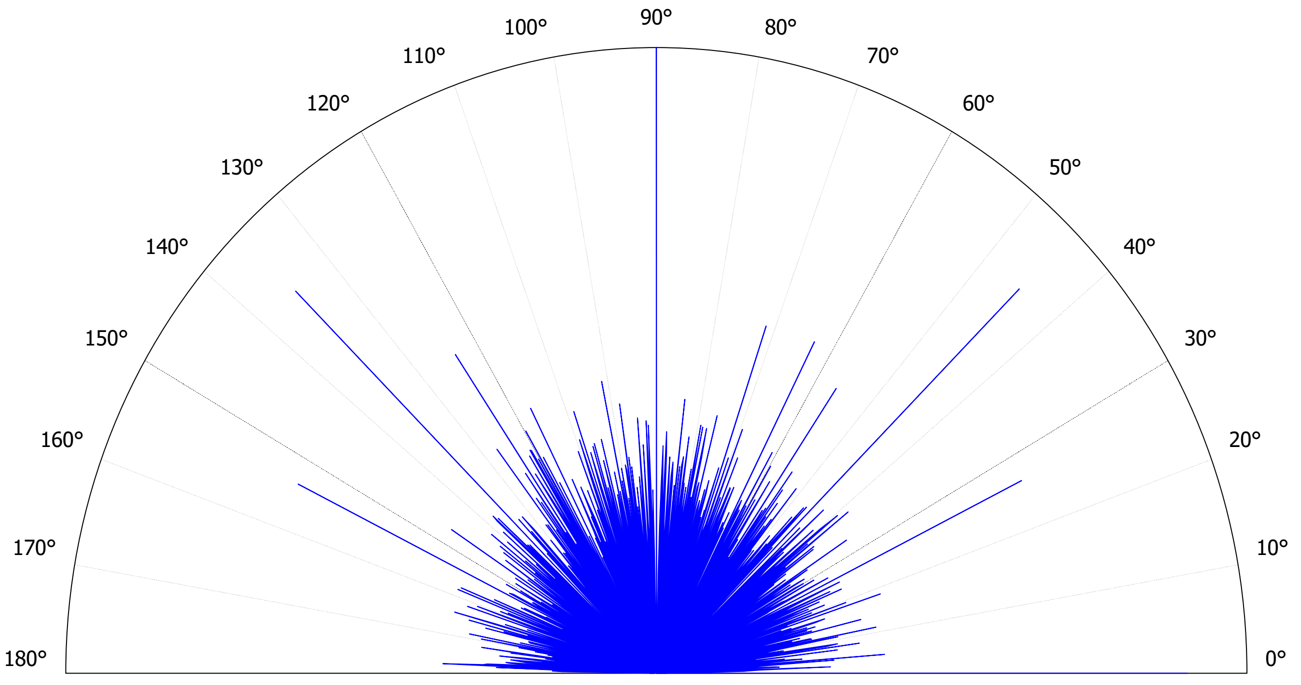

Parameters	Value	Unit
Isotropy	73.8	%
First Direction	90.0	°
Second Direction	0.107	°
Third Direction	45.1	°

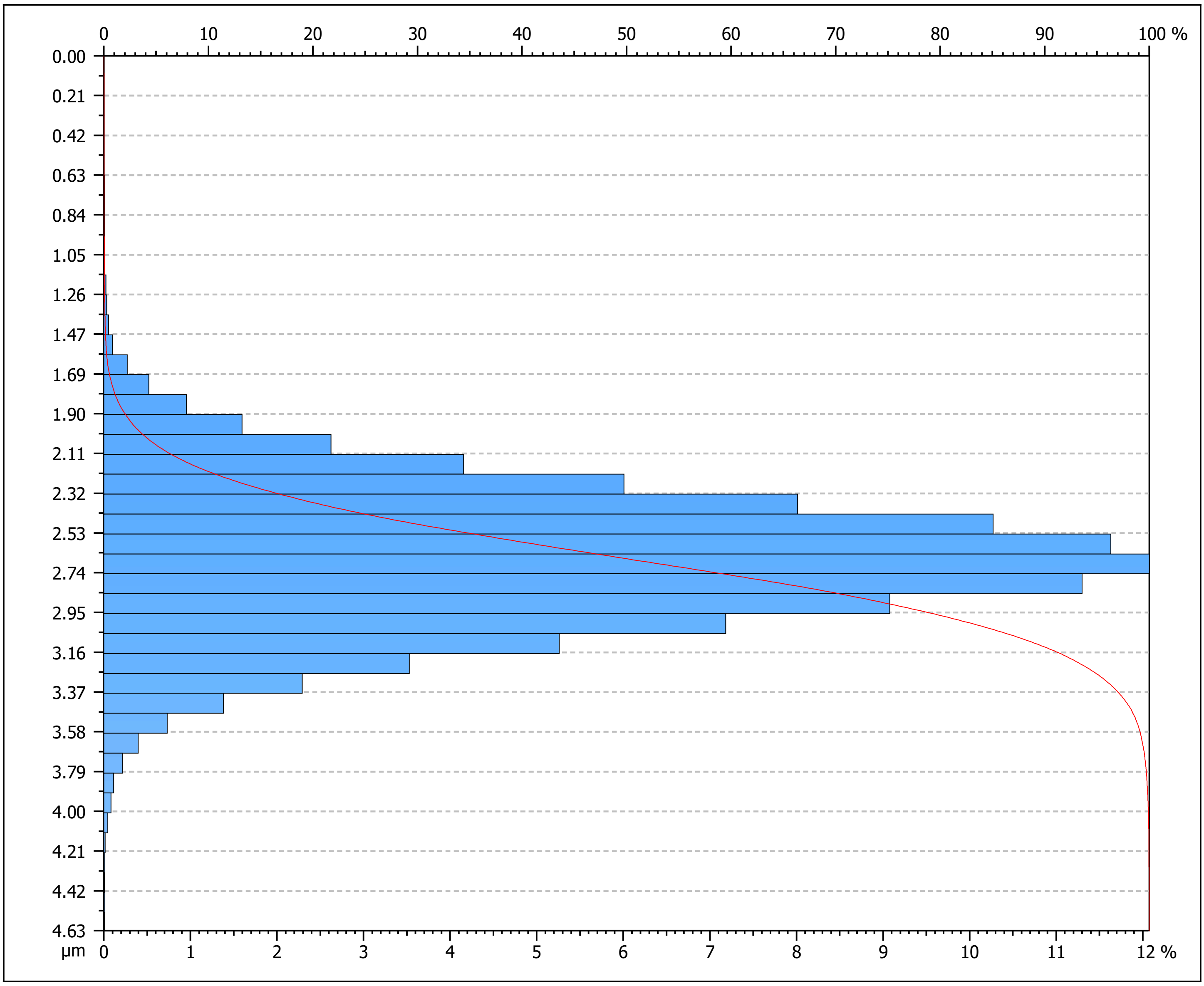

MountainsMap® Premium 7.4.8083
