# Supplementary material for: Discrimination of Surface Topographies Created by Two-Stage Process by Means of Multiscale Analysis
Source: Materials (Basel). 2021 Nov 20;14(22):7044. doi: 10.3390/ma14227044 (PMC8624860; doi:10.3390/ma14227044)
Supplement: Supplementary file 1 [file materials-14-07044-s001.zip › supplementary materials/rose plots and heights distribution/RingB_upper_1 180-198.pdf]

| Identity card | |
|---|---|
| Name: | P4C_boczna_1_1  180-198 > P4C_boczna_1_1  180-198 > P4C_boczna_1_1  180-198 > P4C_boczna_1_1  180-198 |

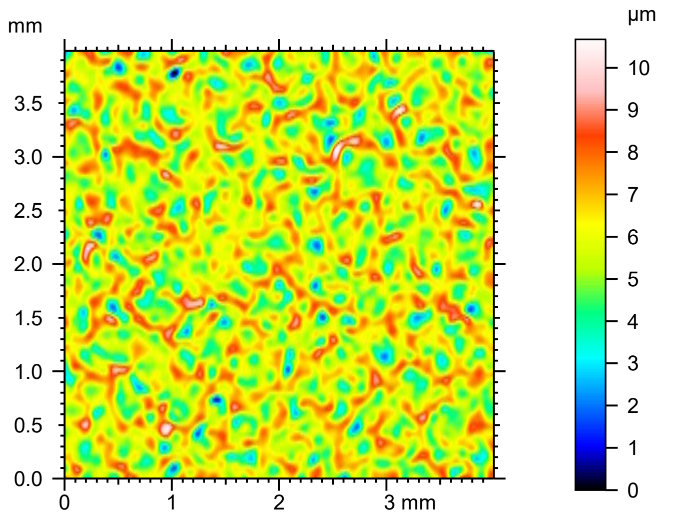


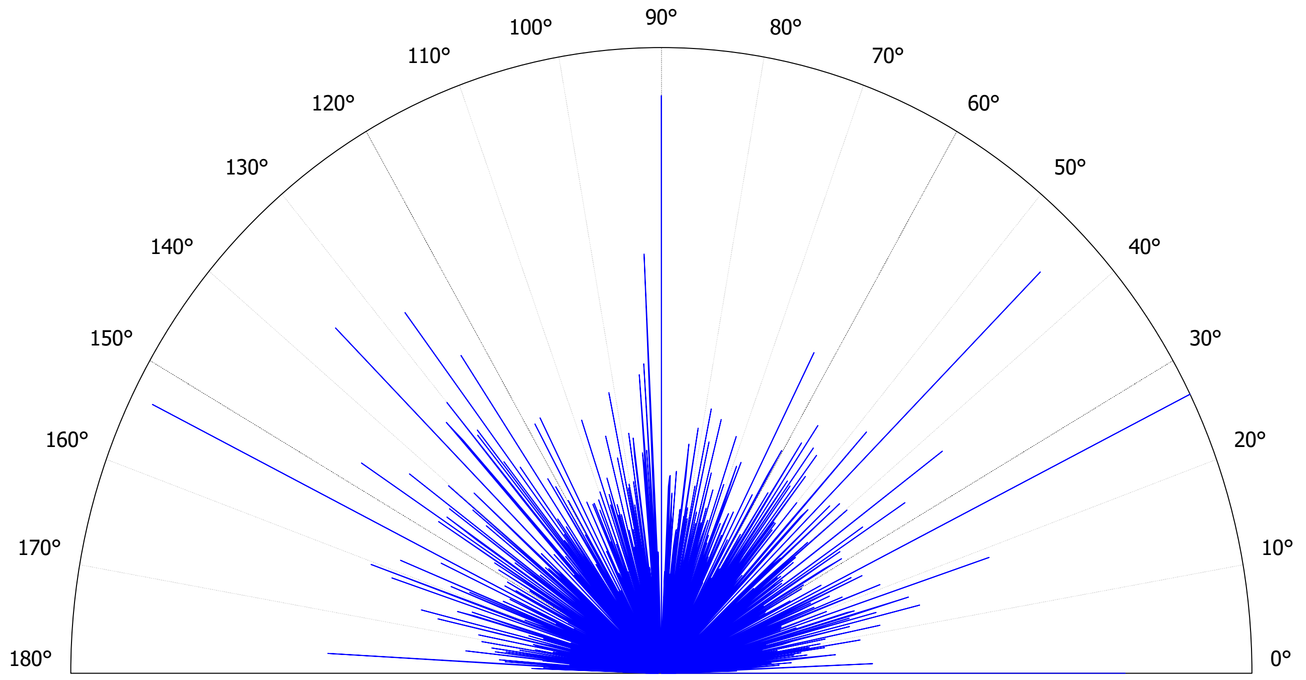


| Parameters | Value | Unit |
|---|---|---|
| Isotropy | 84.3 | % |
| First Direction | 26.5 | ° |
| Second Direction | 153 | ° |
| Third Direction | 90.0 | ° |

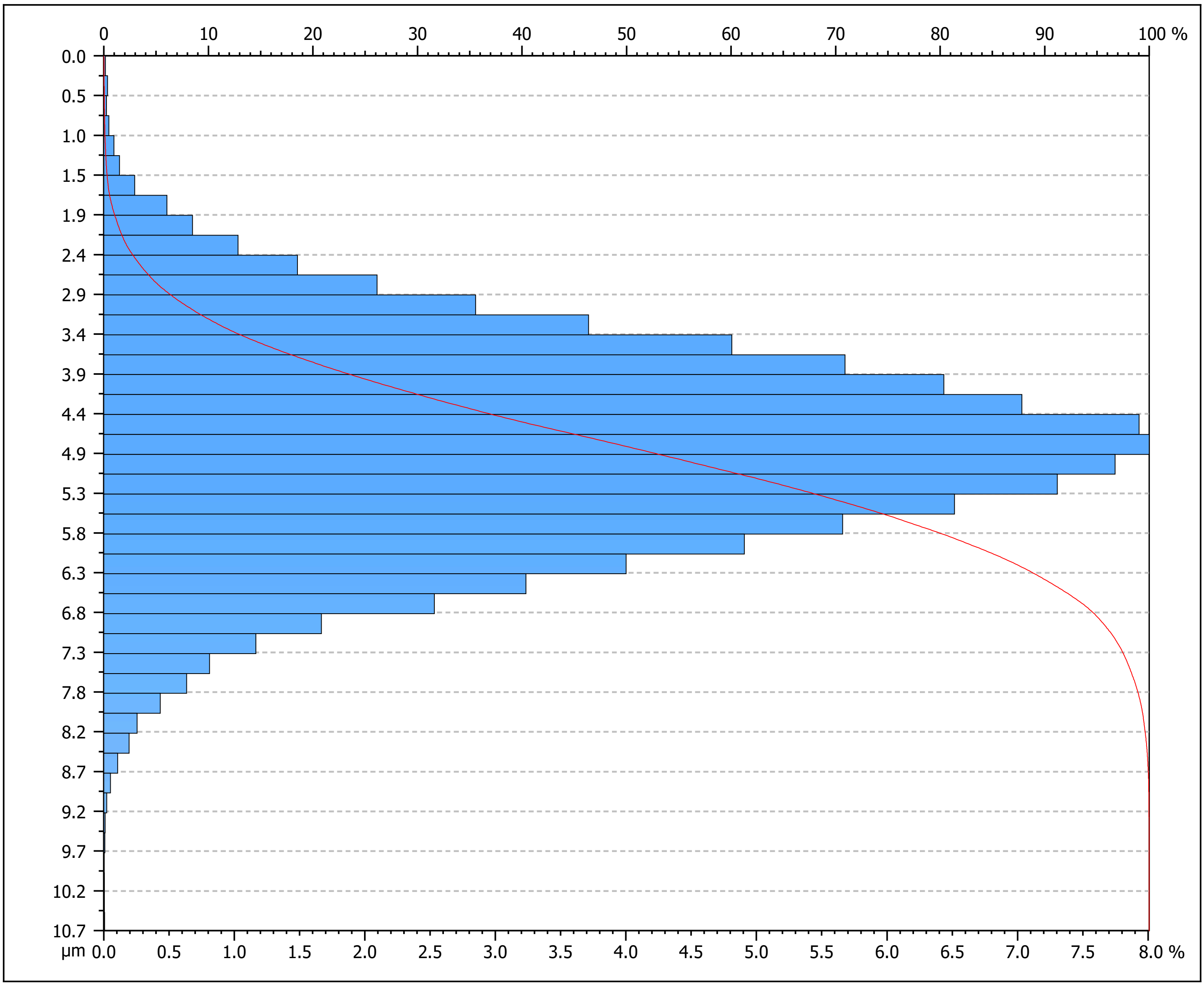


MountainsMap® Premium 7.4.8083
