# Supplementary material for: Discrimination of Surface Topographies Created by Two-Stage Process by Means of Multiscale Analysis
Source: Materials (Basel). 2021 Nov 20;14(22):7044. doi: 10.3390/ma14227044 (PMC8624860; doi:10.3390/ma14227044)
Supplement: Supplementary file 1 [file materials-14-07044-s001.zip › supplementary materials/rose plots and heights distribution/RingB_upper_1 60-160.pdf]

Identity card	
Name:	P4C_boczna_1_1 60-160 > P4C_boczna_1_1 60-160 > P4C_boczna_1_1 60-160 > P4C_boczna_1_1 60-160

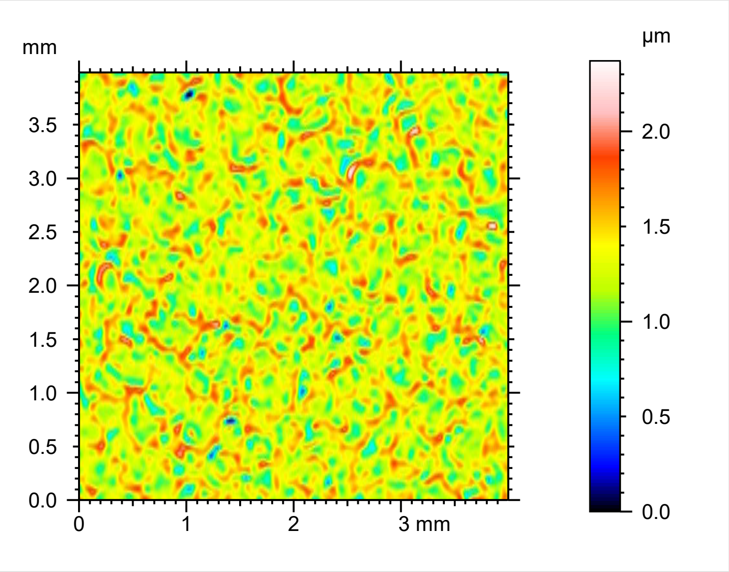

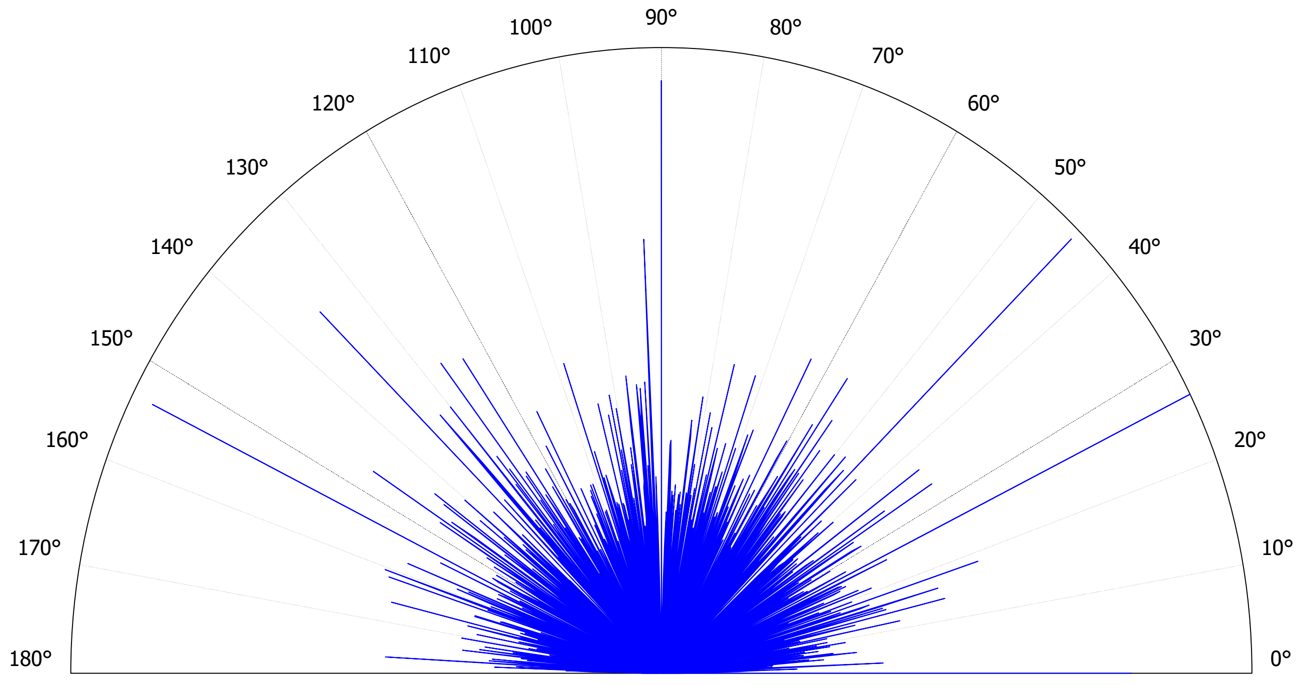

Parameters	Value	Unit
Isotropy	77.6	%
First Direction	26.5	°
Second Direction	45.0	°
Third Direction	153	°

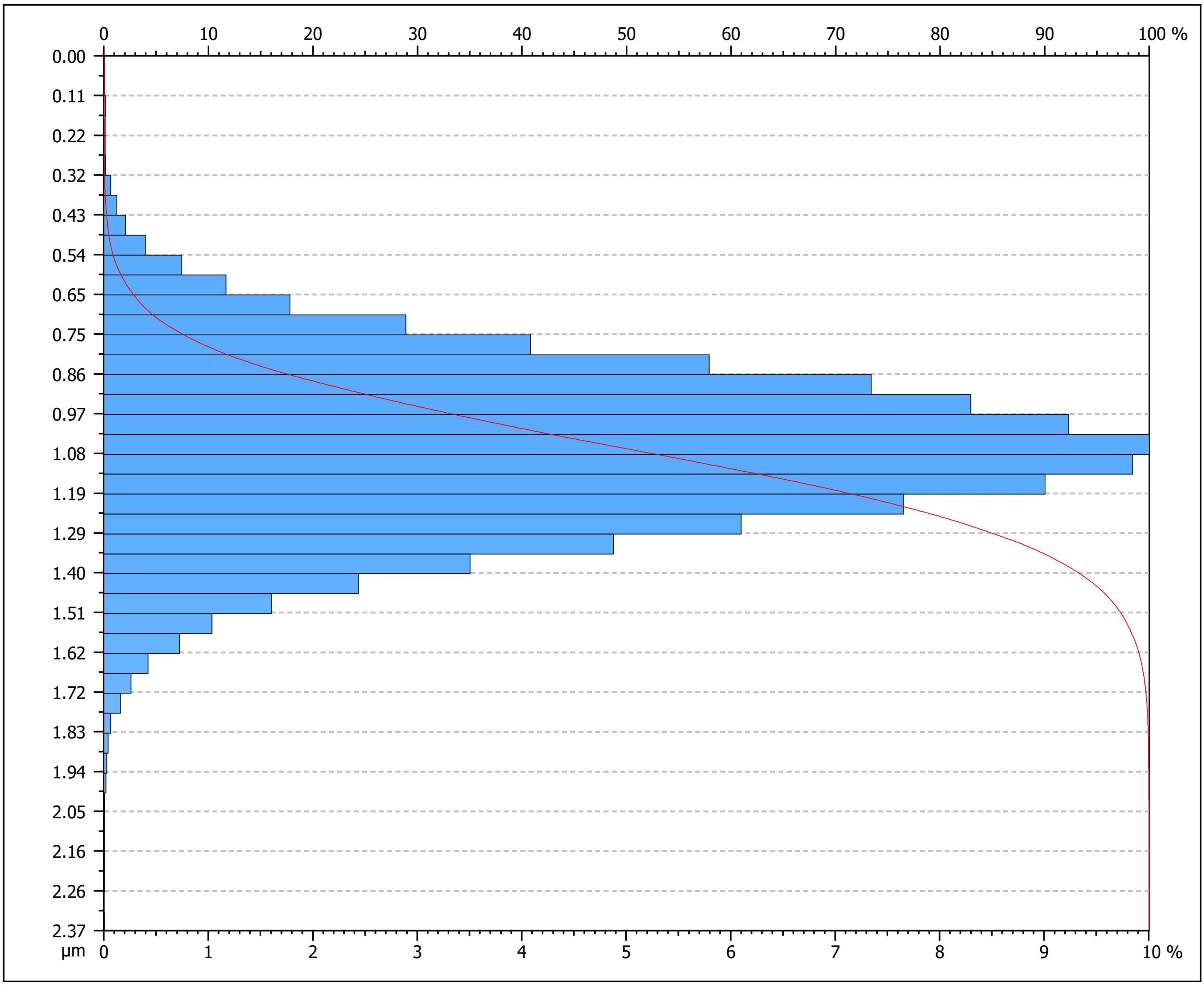

0
10
20
30
40
50
60
70
80
90
100 %
0.00
0.11
0.22
0.32
0.43
0.54
0.65
0.75
0.86
0.97
1.08
1.19
1.29
1.40
1.51
1.62
1.72
1.83
1.94
2.05
2.16
2.26
2.37
µm
0
1
2
3
4
5
6
7
8
9
10 %
